# Marker-Trait Association Analysis of Seed Traits in Accessions of Common Bean (*Phaseolus vulgaris* L.) in China

**DOI:** 10.3389/fgene.2020.00698

**Published:** 2020-06-30

**Authors:** Lei Lei, Lanfen Wang, Shumin Wang, Jing Wu

**Affiliations:** ^1^Institute of Crop Science, Chinese Academy of Agricultural Sciences, Beijing, China; ^2^Institute of Vegetables and Flowers, Chinese Academy of Agricultural Sciences, Beijing, China; ^3^CAAS-CIAT Joint Laboratory in Advanced Technologies for Sustainable Agriculture, Beijing, China

**Keywords:** common bean, population structure, seed weight, seed size, GWAS

## Abstract

Seed weight and seed size are the key agronomic traits that determine yield in common bean. To investigate the genetic architecture of four seed traits (100-seed weight, seed length, seed width, and seed height) of common bean in China, marker-trait association analysis of these seed traits was performed in a nationwide population of 395 common bean accessions using 116 polymorphic SSR markers. The four seed traits were evaluated in six trials across three environments. Seed size varied among the environments. Population structure was evaluated based on SSR markers and phaseolin, which divided the accessions into two main subpopulations representing the two known gene pools. Seed weight and seed size had a strong relationship with population clustering. In addition, in a Genome-wide association studies (GWAS), 21 significantly associated markers were identified for the four seed traits with two models, namely, general linear model (GLM) and mixed linear model (MLM). Some markers had pleiotropic effects, i.e., controlled more than one trait. The significant quantitative trait loci identified in this study could be used in marker-assisted breeding to accelerate the genetic improvement of yield in common bean.

## Introduction

Common bean (*Phaseolus vulgaris* L.) is a crop of major societal importance with high levels of nutrients and dietary protein (Welch et al., 2000; Kutoš et al., 2003; Krupa, 2008; Montoya et al., 2010). Common bean is grown worldwide, with production exceeding 26 million tons (Tg), and China is a large producer of common bean, with the sixth highest production (1 Tg) in the world (FAO, 2018^1^). The species originated in two centers of diversity and, through parallel domestication events, formed two gene pools: the Mesoamerican and Andean gene pools (Gepts et al., 1986; Gepts and Debouck, 1991; Mamidi et al., 2011, 2013; Bitocchi et al., 2013; Gaut, 2014; Schmutz et al., 2014; Rendón-Anaya et al., 2017). As a result of the domestication process, the two gene pools show differences in agronomic traits, such as seed size, seed storage protein (phaseolin) type, bracteole shape, and growth habit, among others. Among these traits, seed size is the most obvious differentiator of the two gene pools, with the Mesoamerican gene pool producing medium [25–40 g 100-seed weight (100SW)] and small seeds (<25 g 100SW) and the Andean gene pool producing medium and large seeds (>40 g 100SW) (Singh et al., 1991a, b, c; McClean et al., 2002, 2004; Broughton et al., 2003; Díaz and Blair, 2006). In addition, seed size is related to phaseolin type (Koenig et al., 1990). China, thought to be the secondary center of genetic diversity for common bean, was reported to include materials from the two gene pools, with seeds being mainly small to medium in size but occasionally large (Zhang et al., 2008; Bellucci et al., 2014). However, compared with soybean, rice and maize (Wang et al., 2015; Xu et al., 2015, 2018; Sangiorgio et al., 2016), common bean (especially Chinese common bean germplasm) has been the subject of few studies on the genetic control of seed traits such as seed weight and seed size.

Seed weight and seed size are the key agronomic traits that determine yield in crops (Xu et al., 2018). It has been reported that yield-related traits such as seed weight and size are typical quantitative traits controlled by a complex of genes since the Danish plant scientist Wilhelm Johannsen concluded that seed size in self-fertilizing beans is influenced by a genetic effect (Johannsen, 1911; Sax, 1923; Motto et al., 1978; Vallejos and Chase, 1991). With the construction of a linkage map based on various types of molecular markers (Vallejos et al., 1992; Nodari et al., 1993; Freyre et al., 1998; Beebe et al., 2006; Hanai et al., 2010; Galeano et al., 2011, 2012) and the completion of genome-wide sequencing of typical common bean materials from the two gene pools (Schmutz et al., 2014; Vlasova et al., 2016), a large number of quantitative trait loci (QTLs) for yield-related traits have been identified (González et al., 2017). To date, a total of approximately 200 QTLs related to seed characteristics have been reported (Nodari et al., 1993; Johnson et al., 1996; Koinange et al., 1996; Freyre et al., 1998; Park et al., 2000; Tar’an et al., 2000; Johnson and Gepts, 2002; Beattie et al., 2003; Guzmán-Maldonado et al., 2003; Blair et al., 2006; Pérez-Vega et al., 2010; Wright and Kelly, 2011; Checa and Blair, 2012; Mukeshimana et al., 2014; Yuste-Lisbona et al., 2014; Qi, 2015; Geng et al., 2017). Among these QTLs, more than 100 have been identified for seed weight, seed length (SL), seed width (SWI) and seed height (SH), and these QTLs are distributed on 11 chromosomes of the common bean genome (Supplementary Table S1). It is worth noting that 16 QTLs are related to at least two of these seed traits (Park et al., 2000; Geng et al., 2017). For example, SL and SH appeared to correspond to a QTL for seed weight (Park et al., 2000). In addition, a study also found the “one cause multieffect phenomenon” for seed traits on Chr02, Chr04, Chr06, and Chr07 of common bean (Geng et al., 2017). In addition, seed size QTLs were mapped near the upper end of linkage groups (LGs) 02 and 06, the lower end of LGs 03, 07, 08, and 10, and the center of LGs 06 and 08 (Park et al., 2000; Blair et al., 2006; Pérez-Vega et al., 2010). Therefore, the markers located in the center of LG B8 and near the upper end of LG B6 could be good candidates for assisted selection for traits related to seed size (Pérez-Vega et al., 2010). However, most of the loci for seed weight or size in these studies were identified in a single environment or using a family group for seed phenotype mapping, which may have caused the parental polymorphism level to be low, in which case the accuracy of QTL mapping would have been affected to some extent.

Important goals in the current study were to (1) investigate the diversity and population structure of 395 common bean accessions with wide geographical distributions in China; (2) carry out marker-trait association analysis in six different environments using general linear model (GLM) and mixed linear model (MLM) approaches, associating phenotypic and genotypic data, with the aim of obtaining stable QTLs for seed traits; and (3) obtain significantly associated markers and provide a theoretical reference for marker-assisted selection (MAS) in the breeding of common bean in China.

## Materials and Methods

### Plant Materials

A total of 395 accessions of common bean were evaluated in this study, including 307 accessions collected from the main production areas of China and 84 accessions introduced from abroad, together with four control genotypes from the Andean and Mesoamerican gene pools. The control genotypes were DRK134 and DRK139, the Andean control genotypes, as well as BAT93 and Turrialba 1, the Mesoamerican controls. The accessions were predominantly landraces; however, the study also included a few modern varieties. All of the materials were selected from the National Crop Gene Bank at the Institute of Crop Science, Chinese Academy of Agriculture Sciences, Beijing, China. The list of accessions and their passport information and source of collection are given in the Supplementary Table S2.

### Phenotypic Evaluation

Field trials were conducted in Harbin (HRB, 45°50′ N and 126°51′ E), Heilongjiang Province, and in Bijie (BJ, 27°18′ N and 105°18′ E), Guizhou Province, in 2014 and 2016, for a total of six environments (2014_HRB, 2015_HRB, 2016_HRB, 2014_BJ, 2015_BJ, and 2016_BJ). Twenty individual plants of each accession were cultivated in two consecutive rows at both locations, and the plots were 4.0 m in length, with 0.5 m between rows.

Five plants from twenty individual plants of each accession were selected randomly, and their seeds were phenotyped. Four quantitative seed traits, namely, (1) 100SW, determined by using 100 dry seeds per plot; (2) SL, defined as the longest dimension of the seed; (3) SWI, measured as the distance between the 2 lateral sides of the seed; and (4) SH, measured as the longest distance from one side to the other at the hilum (Yuste-Lisbona et al., 2014). 100SW, SL and SH were measured by an SC-G automatic seed testing system (Hangzhou WSeen Detection Technology Co., Ltd., China), with 5 replicates of 20 seeds each, which were harvested from 5 individual plants; then, the average of the 5 replicates was calculated. Analysis of variance (ANOVA) was carried out with SPSS 19.0. Pearson correlation coefficients of the traits in each environment were calculated in SAS 9.2 (Cary software, North Carolina SAS Institute Inc., 2004) software.

### SSR Marker and Phaseolin Evaluation

To ensure an adequate sample size for DNA extraction, 10 seeds were randomly selected from each accession, germinated and grown in a greenhouse in Beijing. The first trifoliate leaves of 3-week-old seedlings were collected for total genomic DNA extraction using a CTAB extraction method (Afanador et al., 1993). DNA quality was evaluated with 1% agarose gels, and then DNA was diluted to 10 ng/μl for use in PCRs. PCR amplifications were carried out on an A-300 Fast Thermal Cycler using 96-well plates with 10 μL final reaction volumes that included 1.5 μL of genomic DNA, 1.0 μL of each simple sequence repeat (SSR) primer (Supplementary Material) at a concentration of 2 pmol/L, 4.5 μL of PCR 2 × Master Mix (Mg^2+^, dNTP, Taq polymerase; Beijing Qingke Xinye Biotechnology Co., Ltd.) and 3.0 μL of ddH_2_O. The amplification conditions were as follows: 5 min at 95°C, followed by 35 cycles of 40 s at 95°C, 45 s at 54°C, and 40 s at 72°C and a final extension at 72°C for 10 min. The amplification products were evaluated with silver-stained 8% polyacrylamide gels with 1 × TBE buffer (89 mM Tris, 89 mM boric acid, and 2 mM EDTA). A total of 116 SSR markers distributed over all 11 chromosomes of *P. vulgaris* were used based on the whole genome sequence (Schmutz et al., 2014; Vlasova et al., 2016).

Phaseolin was extracted from 10 dry seeds of each accession, removing the testa and embryo manually from cotyledons before grinding, following the method of Igrejas et al. (2009). The treated seeds were ground into a fine powder in liquid nitrogen. The dry powder (0.1 g) was weighed and placed into 2 mL centrifuge tubes. A total of 1,000 μL of sample extract (1% (w/v) NaCl, 0.4% (w/v) Tris-base, 0.2% (w/v) Tris–HCl, pH of 8.5) and 10 μL of 5% (v/v) β-mercaptoethanol were added for protein extraction. Samples (3.5 μL) were subjected to one-dimensional sodium dodecyl sulfate-polyacrylamide gel electrophoresis (SDS-PAGE) following the method of Ma and Bliss (1978) modified by Igrejas et al. (2009). Electrophoresis was carried out in a 1-mm-thick, 6.38% stacking gel under 40 mA at loading; thereafter, a 12% gel at 75 mA was used until the separation was complete, requiring approximately 5–7 h. The gels were stained with Coomassie brilliant blue R-250. Phaseolin patterns were evaluated with the following standard panel of 14 phaseolin types: S, Sb, Sd, B, M_13_, C, CA, T, PA, To, Ko, CH, H, and H_1_.

### Population Diversity and Structure Analyses

PowerMarker v.3.25 was used to evaluate the number of alleles, gene diversity and polymorphism information content (PIC) for each marker, and clusters were analyzed to construct a dendrogram with PowerMarker^2^ using Nei’s coefficient and the neighbor-joining (NJ) algorithm (Liu and Muse, 2005).

The frequency of each phaseolin type was calculated using Microsoft Office Excel 2016. Two-dimensional principal component analysis (PCA) was performed with GenAlEx 6based on groups of phaseolin types (Peakall and Smouse, 2006).

Population structure and the number of subpopulations (K) were evaluated with STRUCTURE 2.3.4 software (Pritchard et al., 2000; Falush et al., 2007). The assumed number of subpopulations was simulated from *k* = 1 to *k* = 10, and the ideal number of subpopulations was assessed with a burn-in period of 10,000 steps and 20,000 Markov chain Monte Carlo repetitions after the burn-in. The most likely number of subpopulations (K) was determined by examining the optimal △K value (Evanno et al., 2005) in STRUCTURE HARVESTER (Earl and vonHoldt, 2012). A graph (bar plot) of the population structure was generated using the R package pophelper 2.2.9^3^. In addition, to further analyze population structure, the relationships between subpopulations were graphed in three dimensions using NTSYSpc 2.1e (Exeter Software, Rohlf, 2000).

### Association Study

Association analysis between SSR markers and various seed traits was conducted using TASSEL 2.1 (Bradbury et al., 2007). First, a GLM incorporating population genetic structure was fit, with a Q matrix derived from structure analysis as a covariate (GLM + Q). Second, an MLM incorporating a finer-scale relative kinship matrix (K) and population genetic structure (Q) was fit to perform the association analysis (MLM + Q + K), which had more statistical power than the model including only “Q.” Relatedness was determined by calculating the kinship coefficient matrix (K) in TASSEL. Meanwhile, the heritability of seed traits in six enviroments were calculated by MLM model using the same software program.

## Results

### Genetic Variability

First, we evaluated the genetic diversity of the common bean accessions with phaseolin markers. Among these accessions, a total of 11 phaseolin patterns were identified: S, Sb, Sd, B, C, CA, CH, H, PA, T, and To (Supplementary Figure 1). The most frequent pattern was Sb (30.9%), followed by T (21.3%), which were the predominant types in the Mesoamerican and Andean gene pools, respectively. Among these eleven types of phaseolin, the S, Sb, Sd, and B types were Mesoamerican types, while C, CA, CH, H, PA, T, and To were Andean types. Therefore, this variation in protein bands could help distinguish the origins of the accessions. The results indicated that 58.2% of all the accessions belonged to the Mesoamerican gene pool while 41.8% belonged to the Andean gene pool.

Then, we explored the polymorphisms of the same accessions based on SSR markers. A total of 116 SSR markers revealed 917 different alleles, with an average number of alleles per locus of 7.9. The number of alleles identified for each SSR marker varied from 2 (CBS69, CBS170, CBS200, CBS306, P9S39, and CBS369) to 27 (CBS88). The gene diversity of individual SSR markers varied from 0.0051 (CBS200) to 0.9098 (CBS206), with an average of 0.5936 per locus.

### Population Structure

**FIGURE 1:**
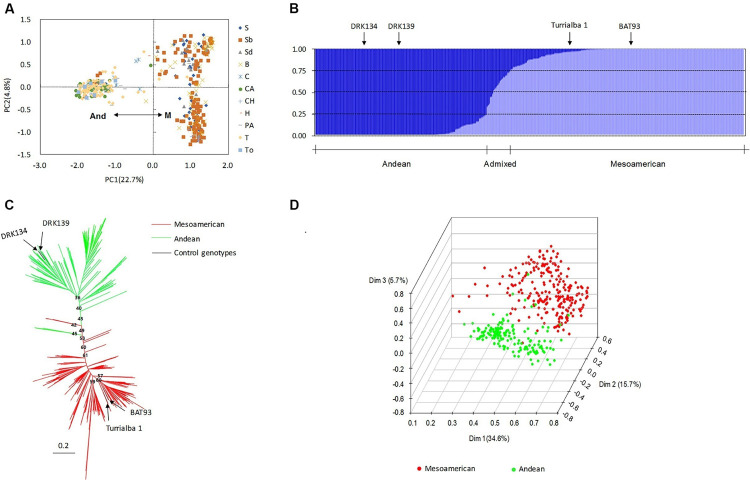
Population structure of 395 accessions. **(A)** Principal component analysis (PCA) of the accessions based on 116 microsatellite loci and phaseolin. The grouping results obtained with phaseolin were almost the same as those obtained with the simple sequence repeat (SSR) markers. And, Andean gene pool; M, Mesoamerican gene pool. **(B)** Population structure of the accessions based on STRUCTURE analysis with *K* = 2. A total of 54.43% of the accessions were assigned to the Mesoamerican gene pool (Q1-values above 0.75), 40.00% were assigned to the Andean gene pool (Q2-values above 0.75), and 5.57% were considered admixed between the two subgroups (Q-values from 0.25 to 0.75). **(C)** Neighbor-joining (NJ) method for determining population structure with Nei’s (1972) genetic distances based on SSR markers. **(D)** Three-dimensional principal coordinate analysis (PCoA) based on all 116 microsatellite markers. DRK134 and DRK139 are the Andean control genotypes, and BAT93 and Turrialba 1 are the Mesoamerican controls.

Population structure is an important covariate used in association analysis to account for differentiation among panel groups and to avoid or minimize type-I errors. It is also important to study population structure in the context of genetic diversity and breeding to examine the genetic composition and relatedness of the individuals within the group. In this article, four methods were used to estimate the number of subgroups. First, PCA based on 116 SSR markers and phaseolin type was performed. Clear subpopulation structure was observed among the individuals, and the two resulting subpopulations corresponded to the Mesoamerican gene pool (M) and the Andean gene pool (And) (Figure 1A). The percentages of genetic diversity explained by the two coordinates of the PCA were 22.7% (PC1) and 4.8% (PC2). Phaseolin types and SSR markers were able to identify the source of the accessions from the two gene pools with almost consistent results. Second, the STRUCTURE software results indicated that the value of Evanno’s △K showed an obvious spike at *K* = 2. This result suggested that the population could be divided into two subgroups, with a few admixed individuals between these two subgroups (Figure 1A). According to the location of the control genotypes in the group, 54.43% of the accessions were assigned to the Mesoamerican gene pool (Q1 values above 0.75), 5.57% were considered admixed between the two subgroups (Q values from 0.25 to 0.75), and 40.00% were assigned to the Andean gene pool (Q2 values above 0.75) (Figure 1B). Third, a NJ dendrogram based on Nei’s (1972) genetic distance also grouped the 395 accessions into two clusters (Figure 1C). Finally, in the three-dimensional principal coordinate analysis (PCoA) based on SSR markers showing the spatial distribution of the 395 accessions, each dimension explained 34.6% (Dim1), 15.7% (Dim2), or 5.7% (Dim3) of the variation (Figure 1D). The three dimensions together explained 56.0% of the total variation present in the data set. Overall, the consistent results from PCA, STRUCTURE analysis, the NJ tree and PCoA confirmed that there were two subpopulations of Chinese common bean germplasm.

**FIGURE 2:**
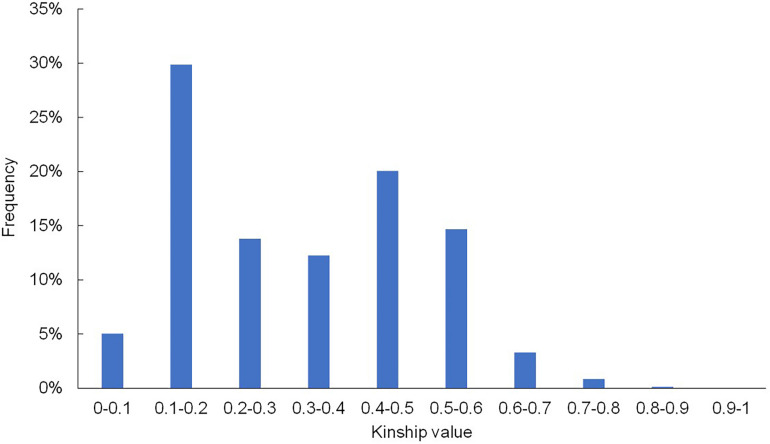
Histogram of the frequency distribution of pairwise relative kinship coefficients. The frequency of kinship values 0–0.5 was greater than 80%, and only 0.15% of the kinship values were above 0.8.

In addition to population structure, the kinship (K) matrix is another important factor for association analysis. The frequency distribution values for relative kinship revealed that the genetic relatedness ranged from 0 to 0.98 (Figure 2), and the average pairwise relative kinship coefficient was 0.323. Pairwise relative kinship values from 0.1 to 0.2 accounted for 29.9% of all kinship coefficients. In addition, kinship values from 0 to 0.5 accounted for more than 80% of all pairwise kinship coefficients. Only 16.957% of the pairwise relative kinship coefficients were greater than 0.5, and only 0.15% of the kinship values were above 0.8. This result suggested that the majority of the 395 accessions were genetically diverse in this study.

### Phenotypic Variability in Seed Traits

**FIGURE 3:**
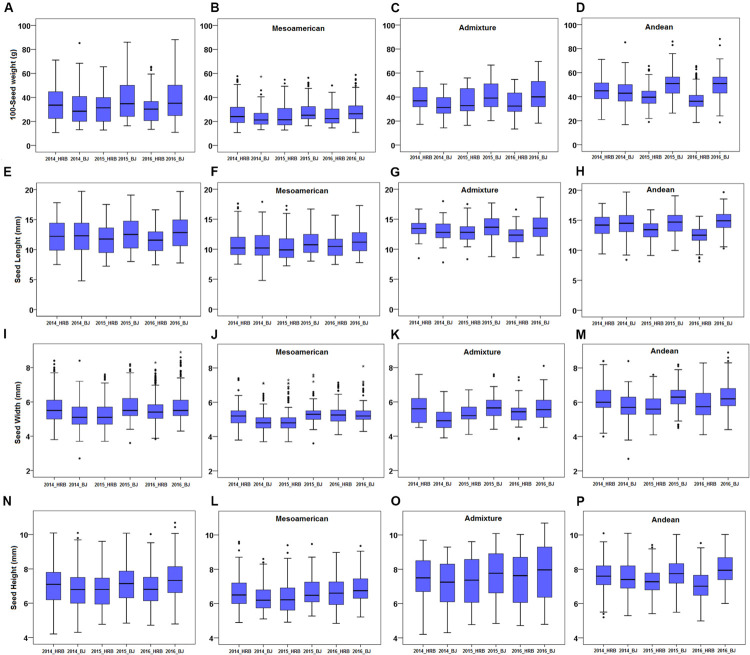
Seed size phenotypes in a common bean population. **(A–D)** 100-seed weight (100SW) of the population: **(A)** 100SW of all accessions, **(B)** 100SW of the Mesoamerican subgroup, **(C)** 100SW of the admixed lines, and **(D)** 100SW of the Andean subgroup. **(E–H)** Seed length (SL) of the population: **(E)** SL of all accessions, **(F)** SL of the Mesoamerican subgroup, **(G)** SL of the admixed lines, and **(H)** SL of the Andean subgroup. **(I–M)** Seed width (SW) of the population: **(I)** SW of all accessions, **(J)** SW of the Mesoamerican subgroup, **(K)** SW of the admixed lines, and **(M)** SW of the Andean subgroup. **(N–P)** Seed height (SH) of the population: **(N)** SH of all accessions, **(L)** SH of the Mesoamerican subgroup, **(O)** SH of the admixed lines, and **(P)** SH of the Andean subgroup. Means were used to assess the differences between environments, and the values are reported as means ± SD. Asterisk indicates outliers.

Differences in growth environments and years affected the weight and size of common bean seeds. Hundred seed weight, SL, SWI and SH were evaluated in six environments, namely, 2014_HRB, 2014_BJ, 2015_HRB, 2015_BJ, 2016_HRB, and 2016_BJ. Each trait had a high degree of variation (Figure 3), especially SWI (Figure 3I–M).

There were significant differences among the environments (Table 1). For the four traits, the differences between years in the same location were smaller than the differences between locations, and there was almost no difference in the same location, such as between 2015_BJ and 2016_BJ or 2015_HRB and 2016_HRB. However, differences between locations were significant. In addition, the heritability of these four traits in each year and environment was analyzed, and the heritability were in the range of 50–70%, so it showed that the environment has a relatively high level of phenotypic plasticity in seed traits. These findings indicated that the environment had a strong impact on traits and the necessity of multipoint phenotypic investigations.

**TABLE 1:** ANOVA analysis of trait in different environments.

Traits	Environment	Mean ± SD	Subgroup			Heritability (%)
			Mesoamerican	Admixture	Andean	
100SW (g)	2014_HRB	34.5113.30Bb	26.128.99BCbc	39.4012.92Aa	45.249.80Bb	61.29
	2014_BJ	31.4613.04Cc	22.826.95De	31.679.74Ab	43.2510.34Bb	53.65
	2015_HRB	31.3511.83Cc	24.459.02CDd	36.1012.18Aa	40.098.67Cc	59.93
	2015_BJ	37.5114.08Aa	28.007.77ABa	40.4413.17Aa	50.0510.43Aa	54.88
	2016_HRB	30.2510.14Cc	24.957.38Ccd	33.6211.54Aa	36.998.95Dd	52.08
	2016_BJ	37.814.29Aa	28.578.40Aa	40.8613.76Aa	49.9311.19Aa	54.82
SL (mm)	2014_HRB	12.32.63Bb	10.842.26BCbc	13.301.94Aab	14.131.86Bb	59.03
	2014_BJ	12.242.77Bb	10.672.19BCc	12.932.21*Aab*	14.292.10ABab	58.72
	2015_HRB	11.682.42Cc	10.382.17*Ccd*	12.892.08Aab	13.271.65Cc	66.56
	2015_BJ	12.572.56ABab	11.122.06ABab	13.582.01Aa	14.411.92ABab	65.12
	2016_HRB	11.472.03Cc	10.581.92Ccd	12.321.90Ab	12.561.56De	64.31
	2016_BJ	12.832.55Aa	11.382.09Aa	13.682.24Aa	14.691.79Aa	68.27
SWI (mm)	2014_HRB	5.610.86ABa	5.190.55Ab	5.660.93ABa	6.170.87Aa	61.08
	2014_BJ	5.210.77Cc	4.840.51Bc	5.010.76Bb	5.750.76Bb	49.75
	2015_HRB	5.280.79Cc	4.910.61Bc	5.350.73ABab	5.770.76Bb	56.63
	2015_BJ	5.730.80Aa	5.290.51Aa	5.730.86Aa	6.330.74Aa	55.30
	2016_HRB	5.530.77Bb	5.260.56Aab	5.450.93ABab	5.910.83Bb	59.28
	2016_BJ	5.730.85Aa	5.310.53Aa	5.690.89Aa	6.310.86Aa	62.74
SH (mm)	2014_HRB	7.061.04Bb	6.610.88Bb	7.481.36a	7.620.89Bbc	24.98
	2014_BJ	6.871.08Cc	6.370.85Cc	7.081.51a	7.510.93BCcd	53.13
	2015_HRB	6.801.02Cc	6.340.86Cc	7.391.42a	7.330.84CDd	59.06
	2015_BJ	7.191.03Bb	6.710.82ABb	7.751.54a	7.760.87Bb	58.21
	2016_HRB	6.870.96Cc	6.630.88Bb	7.431.49a	7.110.87De	59.36
	2016_BJ	7.411.07Aa	6.910.81Aa	7.871.66a	8.040.91Aa	57.34

*Significance of the differences in the table are based on the comparison of differences between different years of the same trait or the comparison among different years of the same sub-group of the same trait. The same traits with different capital letters in the six environments mean have extremely significant differences (p < 0.01), with different lowercase letters mean have significant differences (p < 0.05).*

Furthermore, ANOVA was carried out in different subgroups in the same environment (Table 2). Mesoamerican, admixed and Andean groups were assembled based on the grouping results from STRUCTURE. Among these three subgroups, the Andean group always produced large seeds, while the Mesoamerican group produced small seeds and the admixed group produced medium seeds. These three subgroups showed significant differences, especially the Mesoamerican and Andean subgroups.

**TABLE 2:** ANOVA analysis of trait in different subgroups.

Environment	Subgroup	Trait			
		100SW (g)	SL (mm)	SWI (mm)	SH (mm)
2014_HRB	Mesoamerican	26.12 ± 8.99C	10.84 ± 2.26Bb	5.19 ± 0.55C	6.61 ± 0.88Bb
	Admixture	39.40 ± 12.92B	13.30 ± 1.94Aa	5.66 ± 0.93B	7.48 ± 1.36Aa
	Andean	45.24 ± 9.80A	14.13 ± 1.86Aa	6.17 ± 0.87A	7.62 ± 0.89Aa
2014_BJ	Mesoamerican	22.82 ± 6.95C	10.67 ± 2.19C	4.84 ± 0.51Bb	6.37 ± 0.85Bc
	Admixture	31.67 ± 9.74B	12.93 ± 2.21B	5.01 ± 0.76Bb	7.08 ± 1.51Ab
	Andean	43.25 ± 10.34A	14.29 ± 2.10A	5.75 ± 0.76Aa	7.52 ± 0.93Aa
2015_HRB	Mesoamerican	24.45 ± 9.02Bb	10.38 ± 2.17Bb	4.91 ± 0.61C	6.34 ± 0.86Bb
	Admixture	36.10 ± 12.18Aa	12.89 ± 2.08Aa	5.35 ± 0.73B	7.39 ± 1.42Aa
	Andean	40.09 ± 8.67Aa	13.27 ± 1.65Aa	5.77 ± 0.76A	7.33 ± 0.84Aa
2015_BJ	Mesoamerican	28.00 ± 7.77C	11.12 ± 2.06Bb	5.29 ± 0.51C	6.71 ± 0.82Bb
	Admixture	40.44 ± 13.17B	13.58 ± 2.01Aa	5.73 ± 0.86B	7.75 ± 1.54Aa
	Andean	50.05 ± 10.43A	14.41 ± 1.92Aa	6.33 ± 0.74A	7.76 ± 0.87Aa
2016_HRB	Mesoamerican	24.95 ± 7.38Bb	10.58 ± 1.92Bb	5.26 ± 0.56Bb	6.63 ± 0.88Bb
	Admixture	33.62 ± 11.54Aa	12.32 ± 1.90Aa	5.45 ± 0.93Bb	7.43 ± 1.49Aa
	Andean	36.99 ± 8.95Aa	12.56 ± 1.56Aa	5.91 ± 0.83Aa	7.11 ± 0.87Aa
2016_BJ	Mesoamerican	28.57 ± 8.40C	11.38 ± 2.09Bc	5.31 ± 0.53Bc	6.91 ± 0.81Bb
	Admixture	40.86 ± 13.76B	13.68 ± 2.24Ab	5.69 ± 0.89Bb	7.87 ± 1.66Aa
	Andean	49.93 ± 11.19A	14.69 ± 1.79Aa	6.32 ± 0.86Aa	8.04 ± 0.91Aa

*Significance of the differences in the table are based on the comparison of differences subgroups. The same traits with different capital letters in the six environments mean have extremely significant differences (p < 0.01), with different lowercase letters mean have significant differences (p < 0.05).*

Further exploring the correlation among the traits, we found that 100SW, SL, SWI, and SH were all significantly positively correlated with each other in all six environments (Table 3). Thus, these four seed traits were closely related to each other.

**TABLE 3:** Pearson correlation coefficients for seed traits of common bean.

Year	Traits	100SW	SL	SWI	SH
2014	100SW		0.832***	0.742***	0.787***
	SL	0.794***		0.498***	0.653***
	SWI	0.768***	0.395***		0.731***
	SH	0.666***	0.470***	0.588***	
2015	100SW		0.849***	0.784***	0.830***
	SL	0.822***		0.450***	0.662***
	SWI	0.806***	0.471***		0.697***
	SH	0.903***	0.661***	0.733***	
2016	100SW		0.814***	0.740***	0.837***
	SL	0.777***		0.655***	0.385***
	SWI	0.639***	0.312***		0.683***
	SH	0.755***	0.591***	0.604***	

The data at upper right corner in same year mean trait phenotype were collected from the locations of BJ, and at lower left corner mean trait phenotype were collected from the locations of HRB. ***indicated the significance at the 0.001 level, **indicated the significance at the 0.01 level, *indicated the significance at the 0.05 level.

### Marker-Trait Association Analysis of Seed Traits

Association analysis Genome-wide association studies (GWAS) was conducted using SSR markers and the phaseolin locus (*Phs*) with six phenotypic data sets and two models, namely, GLM + Q and MLM + Q + K. A QTL for *Phs was identified* in LG 07 that spanned a region reported to code for phaseolin seed protein (Nodari et al., 1992; Koinange et al., 1996; Park et al., 2000). *Phs* has been demonstrated to be significantly associated with 100SW, SWI, and SL (Blair et al., 2009). We were interested in confirming the associations identified in previous studies of QTLs for these traits. All the results met the strict threshold of 4.3 × 10^–4^ and had a significance level of 0.05 after Bonferroni correction for both models.

**FIGURE 4:**
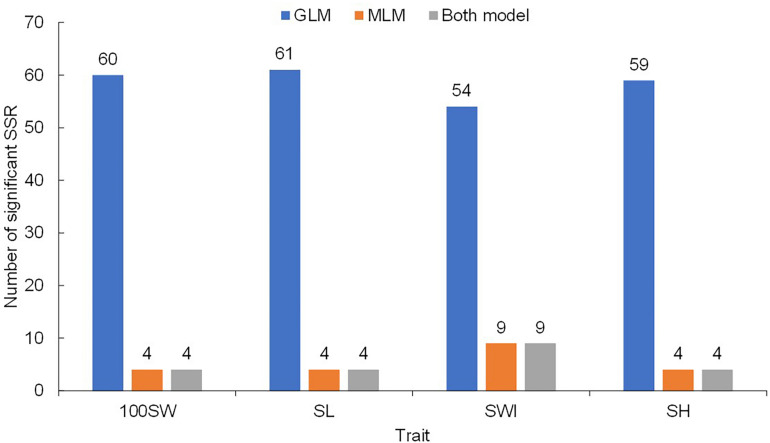
Simple sequence repeat (SSR) markers detected by using different models.

Regarding 100SW, a total of 60 significant SSR markers were detected using the GLM + Q model (Figure 4). Among these markers, 23 were detected in all six environments (Supplementary Table S4). In contrast, only four significant SSR markers were detected with the MLM + Q + K model (Figure 4 and Supplementary Table S5), and these four markers were detected in only a single environment.

Similarly, 61 significant SSR markers were associated with SL in the GLM + Q model (Figure 4), and 44 markers were detected in all six environments (Supplementary Table S4). However, only four significant markers were detected with the MLM + Q + K model, and all were located on Chr05 (Supplementary Table S5). The markers CBS162 and CBS178 were detected simultaneously in two environments with the MLM + Q + K model.

Similarly, 54 significant markers were associated with SWI based on the GLM + Q model, and 12 significant markers were detected in all six environments (Supplementary Table S4). In addition, under the MLM + Q + K model, 10 significant markers were detected (Supplementary Table S5). Among the 10 markers, CBS149 and CBS345 were simultaneously detected in three environments.

For SH, 59 significant markers were detected with the GLM + Q model, and 31 significant markers were detected in all six environments (Supplementary Table S4). However, only four significant markers were detected with the MLM + Q + K model (Supplementary Table S5), and these four markers were detected in a single environment.

**FIGURE 5:**
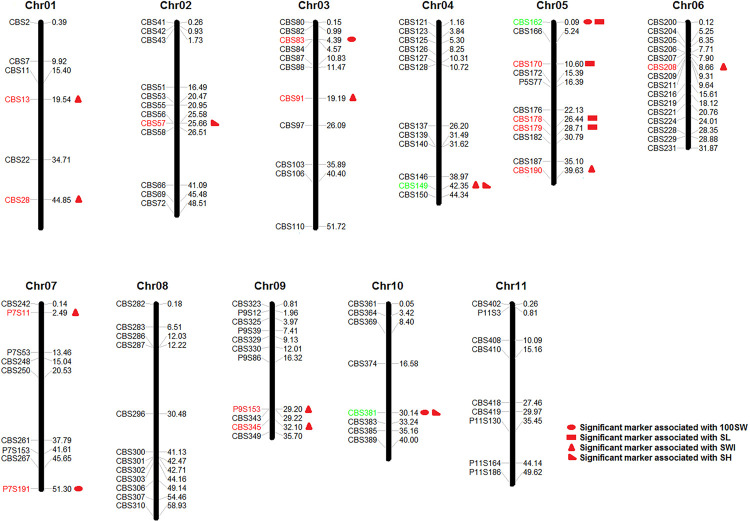
Markers significantly associated with seed traits [100-seed weight (100SW), seed length (SL), seed width (SWI), and seed height (SH)] that were detected with both the GLM + Q model and the MLM + Q + K model. Red markers were significantly associated with a single seed trait, while green markers were significantly associated with multiple seed traits.

In summary, a total of 20 significant SSR markers were detected for four seed traits with both models (Figure 5). Some markers were simultaneously significantly associated with multiple traits, such as the marker CBS162, which was significantly associated with 100SW and SL; CBS381, which was significantly associated with 100SW and SH; and CBS149, which was significantly associated with SWI and SH. This result once again proved that there were correlations among seed traits or that the traits may have overlapping genetic control regions. Moreover, the *Phs* was found to be associated with SWI based on the two models used in our study (Supplementary Table S6).

## Discussion

In this study, we evaluated the genetic diversity of common bean accessions with phaseolin and SSR markers, and phaseolin diversity revealed that the genetic diversity of Chinese common bean was higher than that in other countries or areas (Rodiño et al., 2001, 2006; Blair et al., 2003; Ocampo et al., 2005; Negahi et al., 2014). We even detected several Chinese landraces with phaseolin types such as CH, To, and H, which suggested that the landraces of China have rich variation. Many studies have shown a correlation between phaseolin type and seed weight, seed size, low soil pH, growth habit, precocity and antiparasitic traits (Hartana and Bliss, 1984; Gepts and Bliss, 1986; Koenig et al., 1990; Johnson et al., 1996; Escribano et al., 1998). Therefore, it is very important to identify the phaseolin type of China’s preserved common bean germplasm, which will facilitate yield and insect resistance breeding.

Regarding population structure, Phaseolin and SSR markers from the present study both reveal Chinese common bean germplasms containing two gene pools materials, and the main group of Chinese accessions were of Mesoamerican origin, with fewer of Andean origin. This is different from European countries, where Andean genotypes were more prevalent than Mesoamerican (Raggi et al., 2013; Maras et al., 2015; Caroviæ-Stanko et al., 2017; Pipan and Megliè, 2019). This different distribution of two gene pools genotypes in countries might be related to the time of germplasm introduction, adaptation abilities of germplasm, ecological types, the political regulation within these countries in the past, and consumer preferences. Additionally, distance-based analyses cluster analysis based on microsatellite markers in congruence with the results of phaseolin type analysis up to 88.6% in this study. These results showed that Phaseolin and SSR markers have sufficient power to distinguish between the Mesoamerican and Andean germplasms, and clear about the background of Chinese germplasm. The old and elite common bean landraces of China also has a diverse genetic background based on SSR markers (Supplementary Figure 2). Therefore, it is important to deeply explore the excellent germplasm resources of Chinese common bean and to tap the potential of Chinese common bean to increase yield and improve quality.

In the association analysis, the phaseolin marker *Phs* was significantly associated with SWI (Supplementary Table S6), which agreed with the result from a previous seed size association study (Blair et al., 2006). For the significant SSR markers, we selected a suitable linkage disequilibrium (LD) decay distance of approximately 100 Kb as the confidence interval for screening target trait candidate genes (Rossi et al., 2009). The marker P7S191, which was significantly associated with 100SW, was located near the QTL SW7′ in a previous study (Geng et al., 2017). We also identified significantly associated markers on *Chr03* and *Chr10* that a previous study reported as QTLs for 100SW (Nodari et al., 1993; Johnson et al., 1996; Freyre et al., 1998; Park et al., 2000; Guzmán-Maldonado et al., 2003; Blair et al., 2006). The confidence intervals of the two SSR markers CBS178 and CBS179 on *Pv05*, which were significant for SL, were (24014961, 24215231) and (29909932, 30110144), respectively. Both of these markers were included in the interval of a QTL (20.89–36.47 Mb on *Pv05*) previously reported for SL based on linkage analysis (Geng et al., 2017). These results are more helpful for confirming QTLs location related to seed traits. Nevertheless, the candidate QTLs for seed traits require further verification with improved accuracy such as SNP analysis due to the limitations of this study. Apart from these, we also found the phenomenon of marker one cause multiple effects among seed traits. There have been similar reports in previous studies on seed size of common bean (Blair et al., 2009; Geng et al., 2017). For example, Blair et al. (2009) reported the marker BM183 significantly associated with seed weight, width and length also in both genepools accessions. For this pleiotropism, how to balance the effect of the seed traits is essential to improve the yield. It is proposed that the ratio between the length, width and height of seed be correlated with genetic markers to obtain a trade-off between the two traits (Geng et al., 2017). Inspired by this, we can further obtain high yields by associated the ratios of seed length/width, length/height, width/height and length × width/height, length × height/width with SSR marker to balance each seed trait.

## Conclusion

This study provides a comprehensive picture of genetic diversity and structure of Chinese common bean accessions. Out of 395 accessions, 54.43% were of Mesoamerican origin, 40.00% of Andean, while 5.57% of accessions represented putative hybrids between gene pools. For the most part, the classification of common bean accessions according to phaseolin type analysis was in congruence with the results of distance-based analyses of SSR marker. Based on the population structure, 20 significant SSR markers and 1 significant phaseolin marker were associated with the 4 seed traits by GLM and MLM in this study. Association data of seed traits can be used for common bean breeding, especially in terms of its adaptation to different environments. Thus, by combining and genomic selection, we can effectively select the effective QTL alleles for seed weight and size in breeding populations. The results of this study provide a valuable resource to dissect the role of candidate QTL locus regions of the genome governing seed weight/size in common bean.

## Data Availability Statement

The raw data supporting the conclusions of this manuscript will be made available by the authors, without undue reservation, to any qualified researcher.

## Author Contributions

All authors contributed to the article and approved the submitted version.

## Conflict of Interest

The authors declare that the research was conducted in the absence of any commercial or financial relationships that could be construed as a potential conflict of interest.

## Footnotes

1 http://www.fao.org/faostat/en/

2 http://www.powermarker.net

3 http://royfrancis.github.io/pophelper/
