# Supplementary material for: Marker-Trait Association Analysis of Seed Traits in Accessions of Common Bean (Phaseolus vulgaris L.) in China
Source: Front Genet. 2020 Jun 30;11:698. doi: 10.3389/fgene.2020.00698 (PMC7344293; doi:10.3389/fgene.2020.00698)
Supplement: Supplementary file 1 [file Data_Sheet_1.docx]

**Table S1 QTLs of seed weight and size have been reported**

| Traits | Gene/QTL name | Number | LGs/Chr | Reference |
| --- | --- | --- | --- | --- |
| Seed weight and size | SW | 4 | LGs 01, 03, 04, 07 | Nodari RO ,1992 |
|  | SW | 3 | LGs 01, 07, 11 | Koinange et al., 1996 |
|  | SW, SL, SH | 5,12,19 | LGs 02, 03, 04, 05, 06, 07, 08, 11 | Park et al., 2000 |
|  | QTL1-SM, QTL2-SM, QTL3-SM, QTL4-SM, QTL5-SM | 5 | LGs 01, 02, 03, 04, 05 | Guzmán-Maldonado et al., 2003 |
|  | SW2.1, SW2.2, SW3.1, SW6.1, SW7.1, SW8.1, SW8.2, SW9.1, SW10.1, SW11.1 | 10 | LGs 02, 03, 06, 07, 08, 09, 10, 11 | Blair et al., 2006 |
|  | Swf3.1, Swf4.1, Swf11.1 | 3 | LGs03, 04, 11 | Beebe et al., 2006 |
|  |  | 6 | LGs 05, 06, 08, 11 | Wright and Kelly, 2011 |
|  | SW1^PP^, SW6^PP^, SW9.1^PP^, SW9.2^PP^; SL1.1^PP^, SL1.2^PP^, SL2.1^PP^, SL6^PP^, SL7^PP^, SL10^PP^; SWI2 ^PP^, SWI7 ^PP^, SWI9 ^PP^; ST2 ^PP^, ST9 ^PP^ | 15 | LGs 01,02, 06, 07, 09, 10 | Yuste-Lisbona et al., 2014 |
|  | SW2, SW4, SW6.1, SW6.2, SW7, SW9, SW10, SW6’, SW7’, SW9’, SW11’;  SL2, SL4, SL5, SL6.1, SL6.2, SL7, SL9, SL1’, SL6.1’, SL6. 2’, SL7’, SL9’; SWI2, SWI5, SWI6, SWI8’, SWI9’, SWI11’; SH2, SH3, SH4, SH6.1, SH6.2, SH7, SH8, SH11, SH1’, SH6’, SH7’, SH11’; SLW6, SLW7, SLW8, SLW10, SLW6’, SLW7’, SLW8’;  SLH3, SLH5, SLH6, SLH7, SLH8, SLH9, SLH10, SLH6’, SLH3’ | 57 | Chr01, 02, 03, 04, 05, 06, 07, 08, 09, 10, 11 | Geng et al., 2017 |

**Table S2 395 accessions in wide geographic collection, their germplasm types and the detected type of phaseolin**

| Unified taxation code in China | Common name | Country origin | Province | Germplasm types | Phaseolin |
| --- | --- | --- | --- | --- | --- |
| F0000034 | MAO YAN DOU | China | Shanxi | Landrace | PA |
| F0000045 | YANG CAI DOU | China | Shanxi | Landrace | B |
| F0000049 | JIA LIAN DOU | China | Shanxi | Landrace | B |
| F0000054 | JIAN DOU | China | Shanxi | Landrace | B |
| F0000058 | NIAN DOU | China | Shanxi | Landrace | B |
| F0000089 | HUA CAI DOU | China | Shanxi | Landrace | Sb |
| F0000095 | HUA SE JIAN DOU | China | Shanxi | Landrace | Sd |
| F0000103 | HUA HUI HUI DOU | China | Shanxi | Landrace | Sd |
| F0000117 | TIAN E DOU | China | Inner Mongolia | Landrace | T |
| F0000120 | HUANG YUN DOU | China | Inner Mongolia | Landrace | Sd |
| F0000126 | TIAO HUA MEI BAI LIAN DOU | China | Inner Mongolia | Landrace | CH |
| F0000143 | FAN DOU | China | Inner Mongolia | Landrace | Sb |
| F0000153 | XIAO CAI DOU | China | Inner Mongolia | Landrace | Sb |
| F0000154 | YUE JIN DOU | China | Inner Mongolia | Landrace | Sb |
| F0000161 | XIAO BAI DOU | China | Heilongjiang | Landrace | Sb |
| F0000175 | BAI MAO DOU | China | Heilongjiang | Landrace | B |
| F0000195 | XIAO BAI YUN DOU | China | Heilongjiang | Landrace | Sb |
| F0000199 | JING MI DOU | China | Heilongjiang | Landrace | Sb |
| F0000203 | JING MI DOU | China | Heilongjiang | Landrace | Sb |
| F0000211 | HONG YUN DOU | China | Heilongjiang | Landrace | S |
| F0000217 | FAN DOU | China | Heilongjiang | Landrace | B |
| F0000272 | LONG 70-8014 | China | Heilongjiang | Landrace | CA |
| F0000278 | GAN ZHI MI | China | Heilongjiang | Landrace | B |
| F0000281 | BAI YAO DOU | China | Heilongjiang | Landrace | Sd |
| F0000344 | FAN DOU | China | Heilongjiang | Landrace | Sd |
| F0000385 | HUA FAN DOU | China | Heilongjiang | Landrace | B |
| F0000398 | DA MA CHANG | China | Heilongjiang | Landrace | PA |
| F0000404 | TU ZI TUI | China | Heilongjiang | Landrace | T |
| F0000415 | HUA YAO DOU | China | Heilongjiang | Landrace | Sb |
| F0000440 | JING MI DOU | China | Jilin | Landrace | B |
| F0000442 | BAI WU YA CAI DOU | China | Jilin | Landrace | T |
| F0000444 | BAI WU YA CAI DOU | China | Jilin | Landrace | T |
| F0000445 | BAI TAI | China | Jilin | Landrace | Sb |
| F0000446 | BAI TAI | China | Jilin | Landrace | Sb |
| F0000448 | BAI YUN DOU | China | Jilin | Landrace | T |
| F0000452 | FAN DOU | China | Jilin | Landrace | Sb |
| F0000460 | BAI FAN DOU | China | Jilin | Landrace | S |
| F0000469 | JING MI DOU | China | Jilin | Landrace | S |
| F0000470 | BAI FAN DOU | China | Jilin | Landrace | Sb |
| F0000473 | HONG YUN DOU | China | Jilin | Landrace | Sb |
| F0000474 | CAI DOU | China | Jilin | Landrace | B |
| F0000475 | CAI DOU | China | Jilin | Landrace | Sb |
| F0000485 | TANG DOU | China | Jilin | Landrace | B |
| F0000490 | FAN DOU | China | Jilin | Landrace | PA |
| F0000492 | LUO TANG DOU | China | Jilin | Landrace | PA |
| F0000493 | WU YA CAI DOU | China | Jilin | Landrace | PA |
| F0000494 | WU YA CAI DOU | China | Jilin | Landrace | PA |
| F0000501 | FAN DA DOU | China | Jilin | Landrace | B |
| F0000502 | FAN DOU | China | Jilin | Landrace | C |
| F0000503 | WU JI DOU | China | Jilin | Landrace | Sb |
| F0000509 | HONG DA DOU | China | Jilin | Landrace | PA |
| F0000528 | ZI HUA DOU | China | Heilongjiang | Landrace | T |
| F0000557 | DA HUA HONG (2) | China | Guizhou | Landrace | Sb |
| F0000609 | BAI CAI DOU | China | Yunnan | Landrace | To |
| F0000666 | XIAO ZHA DOU | China | Yunnan | Landrace | T |
| F0000698 | JI YAO ZI DOU | China | Yunnan | Landrace | T |
| F0000867 | CAI DOU | China | Yunnan | Landrace | PA |
| F0000902 | KUAN BIAN DOU | China | Yunnan | Landrace | Sd |
| F0000907 | HE BAO DOU | China | Yunnan | Landrace | Sb |
| F0001006 | QUE DAN DOU | China | Yunnan | Landrace | T |
| F0001089 | YANG CAI DOU | China | Shaanxi | Landrace | S |
| F0001090 | BAI YUN DOU | China | Shaanxi | Landrace | Sb |
| F0001102 | YANG MAN CAI DOU | China | Shaanxi | Landrace | C |
| F0001200 | HUA HONG DOU | China | Shaanxi | Landrace | B |
| F0001244 | MEI JING DOU | China | Shaanxi | Landrace | T |
| F0001292 | YANG CAI DOU | China | Shanxi | Landrace | Sb |
| F0001313 | XIAO BAI LIAN DOU | China | Shanxi | Landrace | Sb |
| F0001318 | BAI CAI DOU | China | Shanxi | Landrace | Sb |
| F0001339 | BAI CAI DOU | China | Shanxi | Landrace | S |
| F0001346 | FEN HONG ZI PI LIAN DOU | China | Shanxi | Landrace | Sb |
| F0001361 | HONG MEI DOU | China | Shanxi | Landrace | B |
| F0001394 | ZI HONG DOU | China | Shanxi | Landrace | Sb |
| F0001416 | HONG CAI DOU | China | Shanxi | Landrace | Sb |
| F0001428 | ZI LIAN DOU | China | Shanxi | Landrace | B |
| F0001433 | XIA HONG DOU | China | Shanxi | Landrace | T |
| F0001434 | HUANG HONG DOU | China | Shanxi | Landrace | T |
| F0001438 | BAI HUANG LIAN DOU | China | Shanxi | Landrace | T |
| F0001445 | HUANG HONG DOU | China | Shanxi | Landrace | T |
| F0001458 | WU SI DOU JIAO | China | Shanxi | Landrace | CA |
| F0001459 | WU SI DOU JIAO | China | Shanxi | Landrace | Sb |
| F0001463 | DAO DI QING | China | Shanxi | Landrace | T |
| F0001471 | HE YUN DOU | China | Shanxi | Landrace | Sb |
| F0001474 | XIAO HUI DOU | China | Shanxi | Landrace | Sb |
| F0001497 | XIAO HUI DOU | China | Shanxi | Landrace | B |
| F0001515 | YANG CAI DOU | China | Shanxi | Landrace | Sb |
| F0001523 | HUI CAI DOU | China | Shanxi | Landrace | Sb |
| F0001542 | HEI HONG DOU | China | Shanxi | Landrace | Sb |
| F0001567 | HEI ZHAN CAI DOU | China | Shanxi | Landrace | Sb |
| F0001568 | LIAN DOU | China | Shanxi | Landrace | T |
| F0001600 | BAI LIAN DOU | China | Shanxi | Landrace | B |
| F0001619 | FEN HUA MEI DOU | China | Shanxi | Landrace | Sd |
| F0001652 | HUA SE DOU | China | Shanxi | Landrace | S |
| F0001663 | HUA DAO DOU | China | Shanxi | Landrace | B |
| F0001670 | MEI DOU | China | Shanxi | Landrace | S |
| F0001671 | SAN KAI DOU JIAO | China | Shanxi | Landrace | B |
| F0001675 | MA QUE DAN | China | Shanxi | Landrace | PA |
| F0001683 | WU JIN DOU JIAO | China | Shanxi | Landrace | H |
| F0001704 | HUA JIN DOU | China | Shanxi | Landrace | Sb |
| F0001705 | MA QUE DAN CAI DOU | China | Shanxi | Landrace | PA |
| F0001717 | BAI XIAO DOU | China | Inner Mongolia | Landrace | S |
| F0001721 | ZI YUN DOU | China | Inner Mongolia | Landrace | T |
| F0001723 | ZI YUN DOU | China | Inner Mongolia | Landrace | Sb |
| F0001731 | FEN LAO LAI SHAO | China | Inner Mongolia | Landrace | Sb |
| F0001743 | LAO LAI BAI | China | Inner Mongolia | Landrace | B |
| F0001744 | FEN LAO LAI SHAO | China | Inner Mongolia | Landrace | T |
| F0001749 | HUI XIAO DOU | China | Heilongjiang | Landrace | S |
| F0001757 | MAO MAO DOU | China | Heilongjiang | Landrace | B |
| F0001760 | TU JIAO | China | Heilongjiang | Landrace | S |
| F0001809 | YU HUA DOU | China | Guizhou | Landrace | Sb |
| F0001823 | DA HONG JIN DOU | China | Guizhou | Landrace | T |
| F0001830 | PENG DOU | China | Guizhou | Landrace | T |
| F0001850 | MU SHU DOU | China | Guizhou | Landrace | T |
| F0001914 | HUI HE ZI | China | Guizhou | Landrace | T |
| F0001924 | YANG XIAO DOU | China | Shaanxi | Landrace | Sb |
| F0001932 | LIU SHI RI ZAO | China | Shaanxi | Landrace | B |
| F0001942 | HONG DOU | China | Shaanxi | Landrace | C |
| F0002032 | SI CHA SI JI DOU | China | Shaanxi | Landrace | T |
| F0002059 | HUA MI SI JI DOU | China | Shaanxi | Landrace | S |
| F0002060 | QUE ER DAN | China | Shaanxi | Landrace | C |
| F0002069 | JIA SI JI DOU | China | Shaanxi | Landrace | Sb |
| F0002073 | HUA MEI DOU | China | Shaanxi | Landrace | Sb |
| F0002080 | QUE ER DAN | China | Shaanxi | Landrace | T |
| F0002117 | BAI DOU ZI | China | Gansu | Landrace | Sb |
| F0002119 | BAI XIAO DOU | China | Gansu | Landrace | Sb |
| F0002120 | BAI XIAO DOU | China | Gansu | Landrace | S |
| —— | A48 | Nepal | —— | Landrace | Sb |
| F0002129 | EX-RICO23 | India | —— | —— | Sb |
| F0002130 | BAT1198 | India | —— | Landrace | B |
| F0002152 | R-5550 | India | —— | —— | To |
| F0002153 | R5350 | Turkey | —— | —— | T |
| F0002157 | BAT896 | India | —— | —— | Sb |
| F0002166 | BAT331 | Turkey | —— | Landrace | B |
| F0002167 | BAT85 | Turkey | —— | —— | S |
| F0002169 | BAT336 | Turkey | —— | —— | S |
| F0002170 | M101 | Turkey | —— | —— | CH |
| F0002171 | A51 | Turkey | —— | —— | S |
| F0002176 | DERINET | France | —— | —— | T |
| F0002177 | HARLAN 9113 | France | —— | —— | PA |
| F0002178 | CANNED | France | —— | —— | PA |
| F0002223 | YANG CAI DOU | China | Shanxi | Landrace | Sb |
| F0002229 | JIE HUANG MEI DOU | China | Shanxi | Landrace | B |
| F0002240 | XIAO ZA DOU | China | Shanxi | Landrace | B |
| F0002262 | QIU HONG DOU | China | Shanxi | Landrace | S |
| F0002275 | QIAN HONG CAI DOU | China | Gansu | Landrace | B |
| F0002276 | HONG CAI DOU | China | Gansu | Landrace | B |
| F0002277 | HONG HEI HUA DOU | China | Gansu | Landrace | Sd |
| F0002305 | DOU | China | Hunan | Landrace | Sb |
| F0002379 | BAI LIAN DOU | China | Inner Mongolia | Landrace | B |
| F0002380 | YANG YAN QUAN | China | Inner Mongolia | Landrace | T |
| F0002388 | CAI DOU | China | Inner Mongolia | Landrace | Sb |
| F0002399 | HONG YUN DOU | China | Inner Mongolia | Landrace | B |
| F0002400 | MIAN DOU JIA | China | Inner Mongolia | Landrace | T |
| F0002419 | CAI DOU | China | Inner Mongolia | Landrace | Sb |
| F0002441 | DA HONG CAI DOU | China | Inner Mongolia | Landrace | Sb |
| F0002461 | HUA YUN DOU | China | Inner Mongolia | Landrace | CA |
| F0002462 | HUA CAI DOU | China | Inner Mongolia | Landrace | T |
| F0002471 | JIA QUE DAN | China | Inner Mongolia | Landrace | T |
| F0002477 | XIAO BAI DOU | China | Jilin | Landrace | Sb |
| F0002481 | BIAN BAI DOU | China | Jilin | Landrace | Sb |
| F0002486 | BAI DOU | China | Jilin | Landrace | B |
| F0002489 | BAI FAN DOU | China | Jilin | Landrace | B |
| F0002493 | HONG YUN DOU | China | Jilin | Landrace | Sb |
| F0002502 | NAI HUA YUN DOU | China | Jilin | Landrace | T |
| F0002503 | BAI YUN DOU | China | Heilongjiang | Landrace | B |
| F0002509 | BAI YUN DOU | China | Heilongjiang | Landrace | B |
| F0002521 | HEI YUN DOU | China | Heilongjiang | Landrace | S |
| F0002523 | HEI YUN DOU | China | Heilongjiang | Landrace | Sd |
| F0002527 | HUA YUN DOU | China | Heilongjiang | Landrace | Sb |
| F0002528 | HUA YUN DOU | China | Heilongjiang | Landrace | S |
| F0002535 | DA MA ZHANG | China | Heilongjiang | Landrace | C |
| F0002537 | CAI DOU | China | Heilongjiang | Landrace | C |
| F0002584 | BAI SI JI DOU | China | Sichuan | Landrace | Sb |
| F0002608 | JI YAO ZI DOU | China | Sichuan | Landrace | C |
| F0002612 | XIAO ZHU YAO DOU | China | Sichuan | Landrace | PA |
| F0002620 | HONG YAO ZI DOU | China | Sichuan | Landrace | T |
| F0002623 | HONG TIAN JI DOU | China | Sichuan | Landrace | To |
| F0002628 | YAO ZI DOU | China | Sichuan | Landrace | To |
| F0002673 | WU HE DOU | China | Sichuan | Landrace | S |
| F0002675 | TU HE YAO ZI DOU | China | Sichuan | Landrace | B |
| F0002683 | YING KE DOU | China | Sichuan | Landrace | To |
| F0002755 | XIAO HEI SI JI DOU | China | Sichuan | Landrace | Sb |
| F0002810 | HEI WEN HUA DOU | China | Sichuan | Landrace | S |
| F0002834 | HUA MEI DOU | China | Sichuan | Landrace | B |
| F0002838 | SI SHI DOU | China | Sichuan | Landrace | T |
| F0002870 | BAI YUN DOU | China | Guizhou | Landrace | Sb |
| F0002972 | HEI ZI SHAN DOU | China | Guizhou | Landrace | T |
| F0002974 | HEI JIN DOU | China | Guizhou | Landrace | Sd |
| F0002990 | HEI AI ZI DOU | China | Guizhou | Landrace | Sb |
| F0002994 | SI JI DOU | China | Guizhou | Landrace | PA |
| F0002995 | SI JI DOU | China | Guizhou | Landrace | T |
| F0003010 | BI HUA DOU | China | Guizhou | Landrace | T |
| F0003067 | BAI YANG DOU | China | Guizhou | Landrace | Sb |
| F0003082 | YING KE JI YAO DOU | China | Guizhou | Landrace | T |
| F0003096 | HONG YUN DOU | China | Guizhou | Landrace | C |
| F0003106 | HONG JIN DOU | China | Guizhou | Landrace | C |
| F0003112 | SHA DOU | China | Guizhou | Landrace | Sb |
| F0003274 | WU HUA DA TIE QUE DOU | China | Guizhou | Landrace | T |
| F0003342 | BAI CAI DOU | China | Gansu | Landrace | Sb |
| F0003344 | DA LI BAI CAI DOU | China | Gansu | Landrace | T |
| F0003346 | LONG FAN DOU YI HAO | China | Heilongjiang | Landrace | T |
| F0003370 | HARLAN 4506 | America | —— | —— | Sb |
| F0003372 | HARLAN 9743 | France | —— | —— | T |
| F0003382 | FA YIN 11 HAO | France | —— | —— | C |
| F0003396 | FA YIN 11 HAO | France | —— | —— | T |
| F0003405 | QIU BAI YUN DOU | China | Hebei | Landrace | Sb |
| F0003407 | HONG YUN DOU | China | Hebei | Landrace | Sb |
| F0003453 | HEI YUN DOU | China | Hebei | Landrace | Sb |
| F0003461 | LAO HU DUN | China | Hebei | Landrace | T |
| F0003497 | JIA CAI DOU | China | Inner Mongolia | Landrace | S |
| F0003503 | BAI LIAN DOU | China | Inner Mongolia | Landrace | Sb |
| F0003514 | HEI LIAN DOU | China | Inner Mongolia | Landrace | B |
| F0003554 | BAI DAO DOU | China | Hubei | Landrace | B |
| F0003567 | HUANG MEI DOU | China | Hubei | Landrace | Sb |
| F0003569 | ZAO CAI DOU | China | Hubei | Landrace | H |
| F0003574 | DOU ER DAO DOU | China | Hubei | Landrace | Sb |
| F0003575 | CONG SHENG SI JI DOU | China | Hubei | Landrace | Sd |
| F0003578 | HUI MI MI | China | Hubei | Landrace | S |
| F0003582 | HUANG MI MI | China | Hubei | Landrace | Sb |
| F0003591 | WU SI JI DOU | China | Hubei | Landrace | Sb |
| F0003624 | SI YUE E | China | Hubei | Landrace | PA |
| F0003626 | XIAO HEI ZI | China | Hubei | Landrace | Sd |
| F0003635 | HEI DOU ZI | China | Hubei | Landrace | B |
| F0003645 | SI JI DOU | China | Hubei | Landrace | Sb |
| F0003673 | HUA SI JI DOU | China | Sichuan | Landrace | To |
| F0003709 | DA BAI DOU | China | Guizhou | Landrace | T |
| F0003711 | BAI JIN DOU | China | Guizhou | Landrace | Sb |
| F0003740 | SI JI DOU | China | Guizhou | Landrace | Sb |
| F0003758 | SI JI DOU | China | Guizhou | Landrace | CA |
| F0003817 | BANG DOU | China | Guizhou | Landrace | T |
| F0003843 | SI JI DOU | China | Guizhou | Landrace | S |
| F0003970 | HEI HUA DOU | China | Guizhou | Landrace | T |
| F0003990 | DA DAN DOU | China | Guizhou | Landrace | T |
| F0003992 | HONG HUA DOU | China | Guizhou | Landrace | T |
| F0004027 | HE CAI DOU | China | Gansu | Landrace | B |
| F0004028 | BAI HUA DOU | China | Gansu | Landrace | Pa |
| F0004188 | JIANG MEI DOU | China | Inner Mongolia | Landrace | Sb |
| F0004216 | JIA CAI DOU | China | Inner Mongolia | Landrace | Sb |
| F0004227 | YUN DOU | China | Inner Mongolia | Landrace | Sd |
| F0004228 | FAN DOU | China | Inner Mongolia | Landrace | Sb |
| F0004233 | YUN DOU | China | Inner Mongolia | Landrace | Sb |
| F0004268 | XIAO YUN DOU | China | Inner Mongolia | Landrace | B |
| F0004273 | AI SHENG HONG | China | Inner Mongolia | Landrace | PA |
| F0004277 | QI CUN LIAN | China | Inner Mongolia | Landrace | Sb |
| F0004313 | YING GUO HONG YUN DOU | Britain | —— | —— | PA |
| F0004321 | ANT49 | CIAT | —— | —— | CA |
| F0004322 | BRB-130 | CIAT | —— | —— | T |
| F0004333 | UNS-27342-51 | CIAT | —— | —— | S |
| F0004334 | LRK32 | CIAT | —— | —— | Sb |
| F0004339 | SEQ1006 | CIAT | —— | —— | T |
| F0004341 | 9249-3 | CIAT | —— | —— | Sb |
| F0004349 | FOI11 | CIAT | —— | —— | T |
| F0004350 | MCD2409 | CIAT | —— | —— | CA |
| F0004356 | BRB-133 | CIAT | —— | —— | C |
| F0004357 | DOR483 | CIAT | —— | —— | Sb |
| F0004374 | DRK139 | CIAT | —— | —— | CA |
| F0004378 | FOT32 | CIAT | —— | —— | T |
| F0004381 | FOT39 | CIAT | —— | —— | T |
| F0004395 | ISB-82-865 | CIAT | —— | —— | T |
| F0004396 | DRK134 | CIAT | —— | —— | CA |
| F0004398 | FOT25 | CIAT | —— | —— | S |
| F0004404 | DOR482 | CIAT | —— | —— | Sb |
| F0004410 | VIVA | CIAT | —— | —— | B |
| F0004412 | Turrialba 1 | Costa Rica | —— | —— | Sb |
| F0004413 | BAT58 | CIAT | —— | —— | Sd |
| F0004523 | HUA SI JI DOU | China | Hubei | Landrace | Sd |
| F0004529 | BAI SI JI DOU | China | Hubei | Landrace | Sb |
| F0004538 | CAO BAI DOU | China | Hubei | Landrace | T |
| F0004558 | HUA FAN DOU | China | Hubei | Landrace | Sd |
| F0004559 | YAN SE CAI DOU | China | Hubei | Landrace | S |
| F0004562 | ZI LIAN DOU | China | Hubei | Landrace | Sb |
| F0004564 | MIAN JIAO DOU | China | Hubei | Landrace | Sb |
| F0004566 | YUAN NAI HUA YUN DOU | China | Hubei | Landrace | CA |
| F0004568 | ZI GU LU PO DOU | China | Hubei | Landrace | T |
| F0004590 | MAN JIA DOU | China | Inner Mongolia | Landrace | T |
| F0004592 | NAI HUA YUN DOU | China | Inner Mongolia | Landrace | T |
| F0004594 | JIN DOU JIAO | China | Inner Mongolia | Landrace | B |
| F0004595 | HEI JIA DOU | China | Inner Mongolia | Landrace | S |
| F0004837 | HEI YUN DOU | China | —— | Landrace | Sb |
| F0004845 | HEI YUN DOU | China | —— | Landrace | S |
| F0004851 | HEI YUN DOU | China | —— | Landrace | Sb |
| F0004852 | HEI YUN DOU | China | —— | Landrace | Sb |
| F0004868 | NAI HUA YUN DOU | China | —— | Landrace | T |
| F0004869 | NAI HUA YUN DOU | China | —— | Landrace | CA |
| F0004882 | NAI HUA YUN DOU | China | —— | Landrace | T |
| F0004883 | NAI HUA YUN DOU | China | —— | Landrace | T |
| F0004886 | NAI HUA YUN DOU | China | —— | Landrace | T |
| F0004890 | NAI HUA YUN DOU | China | —— | Landrace | CA |
| F0004895 | NAI HUA YUN DOU | China | —— | Landrace | T |
| F0004899 | NAI HUA YUN DOU | China | —— | Landrace | T |
| F0004910 | NAI HUA YUN DOU | China | —— | Landrace | T |
| F0004911 | NAI HUA YUN DOU | China | —— | Landrace | CA |
| F0004918 | NAI HUA YUN DOU | China | —— | Landrace | T |
| F0004923 | NAI HUA YUN DOU | China | —— | Landrace | CA |
| F0004926 | NAI HUA YUN DOU | China | —— | Landrace | T |
| F0004936 | SU YUN DOU 1 HAO | China | Jiangsu | Landrace | T |
| F0005003 | GE LI (Gally) | Turkey | —— | —— | PA |
| F0005025 | A0640-1 | China | Yunnan | Modern cultivar | CA |
| F0005026 | A0907 | China | Yunnan | Modern cultivar | CA |
| F0005027 | NV | China | Yunnan | Modern cultivar | Sb |
| F0005028 | NR | China | Yunnan | Modern cultivar | Sb |
| F0005029 | LONG YUN DOU 5 HAO | China | Heilongjiang | Modern cultivar | Sb |
| F0005030 | LONG 22-0579 | China | Heilongjiang | Modern cultivar | Sd |
| F0005031 | YUN FENG 2 HAO | China | Yunnan | Modern cultivar | CA |
| F0005032 | PIN YUN 1 HAO | China | Heilongjiang | Modern cultivar | S |
| F0005033 | PIN YUN 2 HAO | China | Heilongjiang | Modern cultivar | Sb |
| F0005034 | LONG YUN DOU 3 HAO | China | Heilongjiang | Modern cultivar | CA |
| F0005035 | 260205 | China | Yunnan | Modern cultivar | Sb |
| F0005036 | 260216 | China | Yunnan | Modern cultivar | CA |
| F0005038 | 260218 | China | Yunnan | Modern cultivar | CA |
| F0005039 | 260219 | China | Yunnan | Modern cultivar | CA |
| F0005040 | 260220 | China | Yunnan | Modern cultivar | C |
| F0005090 | DA DOU | China | Shaanxi | Landrace | Sb |
| F0005219 | HONG YUN DOU | China | Shaanxi | Landrace | Sb |
| F0005237 | LONG YUN DOU 4 HAO | China | Heilongjiang | Modern cultivar | Sb |
| F0005241 | LONG 23－0001NR | China | Heilongjiang | Modern cultivar | Sb |
| F0005243 | LONG 270240 | China | Heilongjiang | Modern cultivar | C |
| F0005244 | LONG 270267 | China | Heilongjiang | Modern cultivar | CA |
| F0005245 | LONG 270270 | China | Heilongjiang | Modern cultivar | CA |
| F0005246 | LONG 270280 | China | Heilongjiang | Modern cultivar | S |
| F0005248 | LONG 270705 | China | Heilongjiang | Modern cultivar | C |
| F0005249 | LONG 270709 | China | Heilongjiang | Modern cultivar | C |
| F0005253 | HONG YAO DOU | China | Gansu | Landrace | Sb |
| F0005256 | XIONG MAO DOU | China | Gansu | Landrace | PA |
| F0005263 | SI JI DOU | China | Gansu | Landrace | Sb |
| F0005269 | NAI DOU | China | Gansu | Landrace | PA |
| F0005295 | WEN CHANG SI JI DOU | China | Gansu | Landrace | B |
| F0005300 | XIAO HEI YUN DOU | China | Hebei | Landrace | Sb |
| F0005361 | LANG TAI BAI CAI DOU | China | Yunnan | Landrace | S |
| F0005363 | HA SHI CAI DOU | China | Yunnan | Landrace | T |
| F0005420 | HONG JIN DOU | China | Yunnan | Landrace | T |
| F0005453 | A1947 | China | Yunnan | Landrace | C |
| F0005457 | XIAO HUA DOU | China | Yunnan | Landrace | T |
| F0005524 | 60 Dias | Brazil | —— | —— | CA |
| F0005526 | Cubano | Brazil | —— | —— | Sb |
| F0005529 | Paraná 1 | Brazil | —— | Landrace | S |
| F0005534 | Paraná | Brazil | —— | —— | S |
| F0005536 | BR IPA 10 | Brazil | —— | —— | Sd |
| F0005538 | Milionário 1732 | Brazil | —— | —— | Sb |
| F0005540 | Preto Catarinense | Brazil | —— | —— | Sb |
| F0005550 | Jalo Precoce | Brazil | —— | —— | To |
| F0005555 | Novo Jalo | Brazil | —— | —— | CA |
| F0005568 | EMGOPA 201-Ouro | Brazil | —— | —— | Sb |
| F0005574 | Carioca MG | Brazil | —— | —— | Sb |
| F0005575 | Tambó | Brazil | —— | —— | S |
| F0005620 | QIAN KE LIU HAO | China | Hebei | Modern cultivar | S |
| F0005623 | 97-5 JIA DOU WANG | China | Shandong | Modern cultivar | PA |
| F0005624 | GAI LIANG LV FENG | China | Shandong | Modern cultivar | S |
| F0005627 | GAI LIANG JIU LI BAI | China | Heilongjiang | Modern cultivar | Sb |
| F0005628 | YOU BAI TE 518 | China | Shandong | Modern cultivar | Sb |
| F0005629 | SI JI JIA DOU WANG | China | Shandong | Modern cultivar | S |
| F0005631 | DA PENG XIN XING（97-5） | China | Shandong | Modern cultivar | PA |
| F0005644 | ZHONG MAO 918 YUN DOU | China | Shandong | Modern cultivar | S |
| F0005684 | SI JI DOU | China | Yunnan | Landrace | Sb |
| F0005715 | EMGOPA 202 - Rubi | Brazil | —— | —— | Sb |
| F0005716 | Palmital Precoce | Brazil | —— | —— | T |
| F0005717 | Iraí | Brazil | —— | —— | T |
| F0005718 | Eriparsa 1 | Brazil | —— | —— | T |
| F0005719 | IAPAR 57 | Brazil | —— | —— | S |
| F0005721 | Ouro Branco | Brazil | —— | —— | T |
| F0005722 | Callacatlan Frijol | Mexico | —— | Landrace | S |
| F0005725 | YJ009757 | Brazil | —— | —— | To |
| F0005767 | SER4 | Colombia | —— | —— | CH |
| F0005769 | SER6 | Colombia | —— | —— | Sb |
| F0005771 | SER8 | Colombia | —— | —— | Sb |
| F0005784 | SER21 | Colombia | —— | —— | Sb |
| F0005798 | SER35 | Colombia | —— | —— | Sb |
| F0005813 | SEN13 | Colombia | —— | —— | Sb |
| F0005830 | SEN32 | Colombia | —— | —— | Sb |
| F0005831 | SEN33 | Colombia | —— | —— | Sb |
| F0005833 | SEC1 | Colombia | —— | —— | B |
| F0005841 | SEC10 | Colombia | —— | —— | S |
| F0005843 | SEC14 | Colombia | —— | —— | B |
| F0005849 | YIN HONG 4 | Colombia | —— | —— | CH |
| F0005857 | 02A4 | Argentina | —— | —— | T |
| F0005860 | LONG YUN DOU 6 HAO | China | Heilongjiang | Modern cultivar | CA |
| F0005862 | LONG YUN DOU 8 HAO | China | Heilongjiang | Modern cultivar | CA |
| F0005875 | YJ009942 | Brazil | —— | Landrace | Sb |
| F0005878 | YJ010130 | Ecuador | —— | Landrace | PA |
| F0005879 | YJ009916 | Colombia | —— | Modern cultivar | T |
| F0005909 | YJ009966 | Colombia | —— | Modern cultivar | Sb |
| F0005910 | YJ010462 | Colombia | —— | Modern cultivar | S |
| F0005919 | YJ010138 | Peru | —— | Landrace | B |
| F0005924 | YJ009779 | Mexico | —— | Landrace | Sb |
| —— | Manaus No2 | Brazil | —— | —— | Sb |
| —— | Manaus No3 | Brazil | —— | —— | T |
| —— | Manaus No4 | Brazil | —— | —— | T |
| —— | Manaus No5 | Brazil | —— | —— | T |
| —— | HR45 | Canada | Ontario | Modern cultivar | B |
| —— | BAT93 | America | Washington | Modern cultivar | Sb |
| —— | A YUN 2 | China | Xinjiang | Modern cultivar | T |
| —— | DAO DOU | China | Shandong | Landrace | C |
| —— | NAI HUA YUN DOU H | China | Heilongjiang | Landrace | T |
| —— | NAI HUA YUN DOU X | China | Xinjiang | Landrace | T |
| —— | NAI HUA YUN DOU D | —— | introduced from abord | —— | T |
| —— | XIAO HEI YUN DOU | —— | introduced from abord | —— | Sb |

‘——’indicated that the related information is unkown or no detail.

**Table S3 Information of 116 SSR markers**

| Marker | Positon in chromosome | Sequences(5'-3') | | Allele  No | GenotypeNo | Gene  Diversity | PIC |
| --- | --- | --- | --- | --- | --- | --- | --- |
|  |  | Forward | Reverse |  |  |  |  |
| CBS2 | chr1 | CCAATTTGTTGTTGTTGTTG | CAGTTTTCTGCCAATGAAGT | 12 | 12 | 0.7021 | 0.6522 |
| CBS7 | chr1 | TAAAAACAACCAAAATGGCT | GAGAAAATGGCTGACGTG | 5 | 5 | 0.4231 | 0.3564 |
| CBS11 | chr1 | CCATTTAAGAGGAGTGTTCG | TACCAGAGGAAAAGCATACG | 13 | 13 | 0.7454 | 0.7089 |
| CBS13 | chr1 | CATTATCGATCTCGGTTCTC | ATGTCATGCCTCTCTTTCAC | 9 | 9 | 0.6328 | 0.5773 |
| CBS22 | chr1 | CAACCTACCAGCTAGCATCT | AGAGCAAGATACAAAAGGCA | 11 | 11 | 0.7423 | 0.706 |
| CBS28 | chr1 | ACCCGGTCTCTTACTTTCTC | CAGCACCTCTAACTCCACTC | 8 | 8 | 0.4505 | 0.4142 |
| CBS41 | chr2 | ACGTTCCAAAAGTCACAAAC | AATTAGTCGATGCGTGATCT | 7 | 7 | 0.7817 | 0.7542 |
| CBS42 | chr2 | TTGGTAACATACACAATGCAC | AACACATGGAAAAAGGCTAA | 14 | 14 | 0.8023 | 0.7848 |
| CBS43 | chr2 | GAGAATGCTGTTGGAAAGAG | CTCCGAGTAGTATCTGCGTC | 5 | 5 | 0.5154 | 0.4034 |
| CBS51 | chr2 | AATCGAATAAAAGGGAAAGG | ATGCCAAAAGAAGAATTGTG | 11 | 11 | 0.7796 | 0.7504 |
| CBS53 | chr2 | CGACCTTCATATCTCTCAGC | TAGGGCACAGATTCAGTCTT | 7 | 7 | 0.4587 | 0.4174 |
| CBS55 | chr2 | ACGTGCCATAATTTTCAACT | TCATAGAGACGTTGAAACCA | 10 | 10 | 0.7812 | 0.7522 |
| CBS56 | chr2 | TGAATATTCTCTGCCAATACAA | TGCCACACGAGACTAAATTAT | 8 | 8 | 0.7421 | 0.6977 |
| CBS57 | chr2 | TTTGTACAAGACCTGGAGGA | ACAATGCTTCTACATTGCCT | 6 | 6 | 0.5638 | 0.5016 |
| CBS58 | chr2 | AAGGGATGGAAAATCAATCT | CAGAAAAGAAATGAATCCCA | 14 | 14 | 0.848 | 0.8299 |
| CBS66 | chr2 | TCCTTTTCTGATTCAGCACT | CTCCAGTGGCTATTTCTGAG | 9 | 9 | 0.3313 | 0.2954 |
| CBS69 | chr2 | TCAGAGGATAGAAAGTCCGA | CTTTGTCGTGTTGTTCAATG | 2 | 2 | 0.0053 | 0.0053 |
| CBS72 | chr2 | TGGCCTTTTGTTAAGATTGT | TCAAATGAATGGTGATGATG | 8 | 8 | 0.5903 | 0.535 |
| CBS80 | chr3 | TGGTCTTTCACATGAGTTGA | CTGAAACACCAGAGAAGGAG | 6 | 6 | 0.5122 | 0.4315 |
| CBS82 | chr3 | ATGATTCATGAAAACTTGGG | AAGATTCGGATCAAACAATG | 22 | 23 | 0.8517 | 0.8371 |
| CBS83 | chr3 | TGGGTATTGGTTGAAGTTCT | CATCTGCTGTCACACATTTC | 18 | 18 | 0.7783 | 0.7587 |
| CBS84 | chr3 | AAGAAGGGAACAAAGTGGAT | TCTCAACGATGCCTTTTTAT | 9 | 9 | 0.6757 | 0.6272 |
| CBS87 | chr3 | TGTGGAATAGGTAGAGGATTG | TTCAGATAAGGTCATGGAAGA | 12 | 12 | 0.7064 | 0.6666 |
| CBS88 | chr3 | TGTATTCTCACGCTTCCTTT | GTTAGGGCACTATCAGAACG | 27 | 27 | 0.8102 | 0.7983 |
| CBS91 | chr3 | AGTGGAGGAGTTACCCATCT | TTCGGTATCCAATTATTGCT | 16 | 17 | 0.8275 | 0.8079 |
| CBS97 | chr3 | CTTTCTTTTGGTTTCCATTG | CAAGGGTTTGTGAGGTGTAT | 17 | 17 | 0.6138 | 0.5875 |
| CBS103 | chr3 | TTACCCGAATCAAATAGCAT | TTAAAACCACGTGGAAAAAC | 9 | 9 | 0.6137 | 0.5551 |
| CBS106 | chr3 | CCCACTCCTTCTAATTGACA | TAGAGGCTCAGTCTTAACGC | 15 | 15 | 0.7208 | 0.6792 |
| CBS110 | chr3 | GGAAGCCAGGAAAAATAAAT | CCTTTAGGCACACAAACTTC | 11 | 11 | 0.799 | 0.7789 |
| CBS121 | chr4 | AAGGTTCCTTCCCATTTAAC | TGGGTATGAAAAGAAAGACC | 5 | 5 | 0.4092 | 0.3862 |
| CBS123 | chr4 | GATTCTTAACTGCTTGGGTG | TAAATGTGAAACGAAACCGT | 12 | 12 | 0.7078 | 0.6695 |
| CBS125 | chr4 | CGGTGTATATCCAAATCCAT | AATGAATTTATGGGGCTTTT | 9 | 9 | 0.7573 | 0.7196 |
| CBS126 | chr4 | AAACAGCGATTAAAAAGTCG | TCATTGACTCAGCAAAAATG | 6 | 6 | 0.5944 | 0.5241 |
| CBS127 | chr4 | TCTGCGTGAAGTACTCAGTG | TTCCCTTGAGAGGTTAGTGA | 5 | 5 | 0.5415 | 0.4621 |
| CBS128 | chr4 | CCCTAATTAAGGTGTTGCAC | GACGTATGTTGTCCCATCTT | 4 | 4 | 0.6036 | 0.5472 |
| CBS137 | chr4 | ATAAACCTCCGCTAAAACCA | CTTTCCACAAACATTCACG | 5 | 5 | 0.5527 | 0.4968 |
| CBS139 | chr4 | GAGAGAATGCAAAGGCTAGA | CCCATTACAAACTTCAAAGC | 7 | 7 | 0.5932 | 0.5407 |
| CBS140 | chr4 | AGCTTGATCTCCATCAGAAA | TTGGCTTCCCATCTTTAGTA | 5 | 5 | 0.4746 | 0.4087 |
| CBS146 | chr4 | TCCTTTTTCTGTGCAGATTT | GGCTTCACCTACAGACTTTG | 12 | 12 | 0.5265 | 0.5097 |
| CBS149 | chr4 | TGGCTCATGATATTTTGACA | TCCATGAGAGCATAGTGTTG | 7 | 7 | 0.5687 | 0.5185 |
| CBS150 | chr4 | GGAGCAAACATTCTGATGAT | TTGAGTTAAGCACTGCACAC | 4 | 4 | 0.6477 | 0.5875 |
| CBS162 | chr5 | GAAAGTATGGGGTGAAGTTG | TTCATGTCCAACTCATGAAA | 15 | 15 | 0.8385 | 0.8203 |
| CBS166 | chr5 | TCAGTCTCTGGGTTTGTTTC | TCCTCTTGTATCCACACTCC | 5 | 5 | 0.5168 | 0.4244 |
| CBS170 | chr5 | GCAATATCCCAATCTTCTTG | AACGTCATTGATTTGACTCC | 2 | 2 | 0.0643 | 0.0622 |
| CBS172 | chr5 | CCCACTTGTATTTCGTTTTT | TTTCTTCCTCTTTCAGTCCA | 9 | 9 | 0.7307 | 0.688 |
| CBS176 | chr5 | TGGCTTAATTCTAAGTCCCA | TGAAAAGTCATCAACAACCA | 6 | 6 | 0.6468 | 0.5744 |
| CBS178 | chr5 | CCACACACTAACTTGTTTGGTA | TCATTATCGTAGTCTGAATGACA | 7 | 7 | 0.7474 | 0.7052 |
| CBS179 | chr5 | TCTCGTTCAGATGACTCTCC | AGCAAGCTTTGTAGAGTTGG | 5 | 5 | 0.605 | 0.527 |
| CBS182 | chr5 | TCAGTCAGAAAATTCAGCAA | GCAGATATGCATCATTCTACG | 4 | 4 | 0.3162 | 0.2875 |
| CBS187 | chr5 | ACAGTATGTTTGGGTATCCG | TCACACTCTTCTATCCATGAAA | 6 | 6 | 0.6357 | 0.5662 |
| CBS190 | chr5 | GTGGTGCACACAAATTAAGA | TGAATCTCTCGTTACCCATT | 5 | 5 | 0.4195 | 0.3418 |
| P5S77 | chr5 | AACGCGTTAACGATAAGTCT | CTGCAAAAAGGATAAGCACT | 6 | 6 | 0.6709 | 0.5991 |
| CBS200 | chr6 | TCCTCTCTCAGCATTGTCTT | GTGAGAAATTGTGGTCCAGT | 2 | 2 | 0.0051 | 0.0051 |
| CBS204 | chr6 | TTTTCCACAATCCACTTGTA | TGTTGCTTTGTTTGGTAGAA | 3 | 3 | 0.4888 | 0.3768 |
| CBS205 | chr6 | TAATCTAAGAATGCACGGGT | GCAGAAGAAGGTGCTGATAC | 3 | 3 | 0.559 | 0.4968 |
| CBS206 | chr6 | ATTATTACTAGCGGGGGAAT | GCAAATGCACAATAATGGTA | 23 | 23 | 0.9098 | 0.9033 |
| CBS207 | chr6 | AATGTGATCAAAAGGTGGAG | TTTGGTTACTTATGGATGGG | 3 | 3 | 0.2517 | 0.2344 |
| CBS208 | chr6 | TTTCTCGCCTTCTTCATTAG | GTCCCCCTTACAACCTCTAC | 7 | 7 | 0.5484 | 0.4998 |
| CBS209 | chr6 | AACAATGACCTCATCTCAACT | TCCTCTAAGAGACAAAATTGC | 13 | 13 | 0.7285 | 0.6904 |
| CBS211 | chr6 | TGTAATTCATGACGTGGAGA | TGCAGGAAACTATGTAGGTGT | 6 | 6 | 0.6079 | 0.5451 |
| CBS216 | chr6 | CTTAGATGAATGCAAAAGGG | TGTCACTCTTACCCAATTCC | 8 | 8 | 0.4937 | 0.4253 |
| CBS219 | chr6 | GAAGTGAAGCTCAGAAATGG | GGAATATTAGTGGTGACGGA | 8 | 8 | 0.7664 | 0.7273 |
| CBS221 | chr6 | ATTACTCGGGGATTTTAAGG | GATTATGGGTCAGCTGATGT | 7 | 7 | 0.599 | 0.5367 |
| CBS224 | chr6 | TCCTTTTCTCCAACATGAAA | TGAGGATGAATATCAGTACGAGT | 3 | 3 | 0.5047 | 0.3854 |
| CBS228 | chr6 | GGGAACCAAGACACAGATAA | ACCGCATGCTAAGAATAAAG | 5 | 5 | 0.6255 | 0.5654 |
| CBS229 | chr6 | TAGCAACAACGTACTGGACA | AGGGTCTGATTGGAAGAACT | 6 | 6 | 0.749 | 0.704 |
| CBS231 | chr6 | TACACAGGAACCAGTGCATA | GTTAAATTTGAGTCGGATCG | 4 | 4 | 0.5002 | 0.4044 |
| CBS242 | chr7 | CTGAGAGACTCCAACTCCAG | GAGGAGAGAAAGGTTGGAAT | 12 | 12 | 0.7044 | 0.6592 |
| CBS248 | chr7 | AGTGCAGCAGGTAATCATTT | AATGATAAAGACCTCGTCCC | 7 | 7 | 0.657 | 0.6201 |
| CBS250 | chr7 | TGAATTGGAATTGATTAGTGG | GCTAAGACACGTGCTAAGAGA | 15 | 15 | 0.8786 | 0.8663 |
| CBS261 | chr7 | GGAGTGTAGTTAGTACCGCTG | GAACTAATTTCTATGTGTGCCTT | 4 | 4 | 0.5393 | 0.4345 |
| CBS267 | chr7 | TTCCGTTAATTCTCAAGGTC | GCACAAGATTTGTAACATTCC | 7 | 7 | 0.5998 | 0.5215 |
| P7S11 | chr7 | AAGAAACTCCGAAGACAACA | ACCATGTGCCAAATAGATTC | 4 | 4 | 0.5016 | 0.389 |
| P7S53 | chr7 | CAAACCATTTAGCAGTTTCC | ACACATTTACCACTCCCAAG | 4 | 4 | 0.2857 | 0.2671 |
| P7S153 | chr7 | ATGAGTCGCAAAAGTTCATT | CTAACCGTCATTGGATTCAT | 6 | 6 | 0.6194 | 0.5559 |
| P7S191 | chr7 | TATCACATGCTTTCCATCAA | AAAGGTTGAGACACCTGAAA | 9 | 9 | 0.5521 | 0.5004 |
| CBS282 | chr8 | TCCTTTGGAAGAGAATCAAA | GAGTGAAGAGAAACAGGTGC | 4 | 4 | 0.6235 | 0.5458 |
| CBS283 | chr8 | GTTTGGTGAAATACTTGGGA | TGCTTCCTTTTTCTGTCAAT | 3 | 3 | 0.5235 | 0.4106 |
| CBS286 | chr8 | ATTTAATTCCCCCAAGAAAG | ATGTTTGCCATTTTGACTCT | 8 | 8 | 0.6744 | 0.6199 |
| CBS287 | chr8 | GTTTCGTGCATTCTTCTTCT | AGAAACCGTTAACGTGAAAA | 18 | 18 | 0.8422 | 0.8231 |
| CBS296 | chr8 | GTTTTCTGCAACTTGGAAAC | TCTGATAACGAGGCTGAAAT | 9 | 9 | 0.7782 | 0.7426 |
| CBS300 | chr8 | CGACAAATCAAATTTTCCAT | AAGTTCCACTACAAGGCAGA | 3 | 3 | 0.3721 | 0.3049 |
| CBS301 | chr8 | ACCCATTCTAGCACTTTCAA | ATCAAAATCAAAATGTTGGG | 4 | 4 | 0.246 | 0.2195 |
| CBS302 | chr8 | TCTCTTATCACAAATTGTCACC | AAGGTATTGTGCCATTCATC | 14 | 14 | 0.8438 | 0.8242 |
| CBS303 | chr8 | AATTCCAACATTTGTGCTTT | TTGTTGTACCCACAGGAAAT | 5 | 5 | 0.6146 | 0.5363 |
| CBS306 | chr8 | TCATCATCATGGTCACTGTC | CTGTTTTCTTGTGGGATAGC | 2 | 2 | 0.0104 | 0.0103 |
| CBS307 | chr8 | AACAGCTTCATTAGCAGCTC | AACCCTGAAGTAGAGGAAGG | 4 | 4 | 0.0666 | 0.0654 |
| CBS310 | chr8 | GCTACCATTATGGCAACATT | ATTGTCATTCGCATTTCTTT | 6 | 6 | 0.7562 | 0.7199 |
| CBS323 | chr9 | CTTAGGTTCTTGGTCGTTTG | AATTGGAGAAGAAGATGCAA | 9 | 9 | 0.7017 | 0.6557 |
| CBS325 | chr9 | GTTAATGTCAAGCTTTTGGG | CTTTATCCATGTTTGGAGGA | 3 | 3 | 0.5126 | 0.3938 |
| CBS329 | chr9 | GATGAGGGAGATGAGATGAA | GAAGTGACCGAGACTGAGAG | 8 | 8 | 0.591 | 0.5329 |
| CBS330 | chr9 | TGTATTTTACAGTGCGAACG | CACGCCATACAATTACTGAA | 4 | 4 | 0.5979 | 0.5328 |
| CBS343 | chr9 | GATGATCAATGCCAAGAAAT | GTGATTTGCATTTTGTGATG | 11 | 11 | 0.7359 | 0.6949 |
| CBS345 | chr9 | GAGTTGCACTCTCGCATAAT | GGTCACCCCATAGCTTATTT | 8 | 8 | 0.7179 | 0.6827 |
| CBS349 | chr9 | GCACACTTTCATGGTTGATT | GCGTGTGTCTGTTTGTGTAG | 8 | 8 | 0.5693 | 0.487 |
| P9S12 | chr9 | TCTCTTCTTCCACTTCCTCA | TTGAAGAGAAACCCTTGAGA | 9 | 9 | 0.6403 | 0.5788 |
| P9S39 | chr9 | ATTGCTGCAAAATTCTTTGT | CAACTCCGTTTCTCATTCTC | 2 | 2 | 0.1314 | 0.1227 |
| P9S86 | chr9 | CCGATCATAATAACCCTTCA | AAAGAAACATATGGTGGTGC | 5 | 5 | 0.5253 | 0.4247 |
| P9S153 | chr9 | CAGATCTTAACCTCTGCACC | CACACCGCTAAATTTTCTTC | 10 | 10 | 0.7388 | 0.6947 |
| CBS361 | chr10 | GAAGACGTGTTTCTCTTTCG | AAACTCTTCCCAAAATGACA | 7 | 7 | 0.6838 | 0.6482 |
| CBS364 | chr10 | GCTGAAAGATGAAAGTTTGC | AACCTTGGAACACTCTTGAA | 11 | 11 | 0.6565 | 0.6062 |
| CBS369 | chr10 | AAAAGGACAACACCTCACAC | CTTCCTCGTCTTCTTCCTCT | 2 | 2 | 0.4214 | 0.3326 |
| CBS374 | chr10 | TGCGGATTATTTTAAAGAGC | AGAGCAAAATCCAACAAAGA | 7 | 7 | 0.7503 | 0.7155 |
| CBS381 | chr10 | GTTTTGGTTTTCTGCTATGG | GCTACCGTATAAGGTGATCG | 15 | 15 | 0.7576 | 0.7262 |
| CBS383 | chr10 | TCTCTTCTCTCTCCGATCAA | AGATTTGGATTCGTTTCGTA | 5 | 5 | 0.592 | 0.5064 |
| CBS385 | chr10 | TTAACCAGAATCATGCGTTT | CAACAAAAACATCAGCTCAA | 4 | 4 | 0.5247 | 0.4171 |
| CBS389 | chr10 | ATGAGAGTTGTGTTGGGAAG | CTCCTCTTTCCATTTTTGTG | 7 | 7 | 0.6795 | 0.6178 |
| CBS402 | chr11 | AGAAATGCATGACTCTCCAC | GCTATGTCAAAACTTCCCAG | 7 | 7 | 0.6278 | 0.552 |
| CBS408 | chr11 | GCTTCGGTCGCTAAATATAA | TTCAAAGTGTATGTGATCCTTC | 13 | 13 | 0.8037 | 0.7757 |
| CBS410 | chr11 | GAATATTAGTTGGGGCTTCC | TCGCTTGTTGATTTTATTCC | 5 | 5 | 0.5278 | 0.472 |
| CBS418 | chr11 | GGTTGGAAAACTATGTCGAA | AAGATTGCATTTGTCGTTTT | 4 | 4 | 0.533 | 0.4346 |
| CBS419 | chr11 | GTCCCTACATTTCAGGTCAA | TATGTTCTCGACATCAACCA | 5 | 5 | 0.5571 | 0.4869 |
| P11S3 | chr11 | GTTTAAATCAATGTGCCGAC | AGCTTGTATCAAGGAATCCA | 3 | 3 | 0.2719 | 0.239 |
| P11S130 | chr11 | TGGTGACAAATCTTGTGAAA | GGTCAAAATCCACTAAACCA | 13 | 13 | 0.7371 | 0.7056 |
| P11S164 | chr11 | CAATTTACACGATCTTTTGACA | TTTAGGCTACACGATAAATGA | 7 | 7 | 0.7713 | 0.7391 |
| P11S186 | chr11 | AAAATTTTGCTTCCACTTCA | TAACAAACAAATGTCAAGCG | 4 | 4 | 0.5026 | 0.3909 |
| Mean |  |  |  | 7.905 | 7.922 | 0.5936 | 0.5426 |

**Table S4 Significantly associated markers for seed traits identified with GLM+Q model in all six environments**

| Marker name | Chromosome | 100SW | SL | SWI | SH |
| --- | --- | --- | --- | --- | --- |
| CBS7 | chr1 | * | * |  | * |
| CBS11 | chr1 | * | * |  | * |
| CBS13 | chr1 |  | * |  | * |
| CBS28 | chr1 |  |  | * |  |
| CBS41 | chr2 |  | * |  | * |
| CBS42 | chr2 | * | * | * | * |
| CBS51 | chr2 |  |  |  | * |
| CBS55 | chr2 |  |  |  | * |
| CBS57 | chr2 | * |  |  | * |
| CBS58 | chr2 |  | * |  |  |
| CBS72 | chr2 |  | * |  |  |
| CBS87 | chr3 |  |  |  | * |
| CBS88 | chr3 |  | * |  | * |
| CBS91 | chr3 |  |  | * | * |
| CBS103 | chr3 |  |  | * |  |
| CBS106 | chr3 |  | * |  |  |
| CBS125 | chr4 |  |  |  | * |
| CBS139 | chr4 |  | * |  |  |
| CBS149 | chr4 |  |  | * | * |
| CBS162 | chr5 | * | * |  | * |
| CBS170 | chr5 |  | * | * |  |
| CBS178 | chr5 | * | * | * | * |
| CBS179 | chr5 | * | * |  | * |
| CBS182 | chr5 |  | * | * | * |
| CBS187 | chr5 | * | * |  | * |
| CBS190 | chr5 | * | * |  | * |
| P5S77 | chr5 | * | * |  | * |
| CBS205 | chr6 | * | * |  |  |
| CBS206 | chr6 | * | * |  | * |
| CBS209 | chr6 |  | * |  |  |
| CBS211 | chr6 | * | * |  |  |
| CBS219 | chr6 | * | * |  | * |
| CBS228 | chr6 |  |  | * |  |
| CBS229 | chr6 | * | * |  | * |
| CBS248 | chr7 |  | * |  |  |
| CBS261 | chr7 |  | * |  |  |
| P7S153 | chr7 | * | * |  |  |
| P7S191 | chr7 |  | * |  | * |
| CBS282 | chr8 | * | * |  | * |
| CBS286 | chr8 |  | * |  |  |
| CBS287 | chr8 |  | * |  |  |
| CBS296 | chr8 | * | * |  | * |
| CBS302 | chr8 | * | * |  |  |
| CBS303 | chr8 | * | * |  |  |
| CBS310 | chr8 |  | * |  |  |
| CBS323 | chr9 | * | * |  |  |
| CBS325 | chr9 |  | * |  |  |
| CBS329 | chr9 |  | * |  |  |
| CBS343 | chr9 |  | * |  |  |
| CBS345 | chr9 |  |  | * | * |
| P9S12 | chr9 |  | * |  |  |
| P9S153 | chr9 |  | * | * | * |
| CBS364 | chr10 |  |  |  | * |
| CBS381 | chr10 | * | * |  | * |
| CBS383 | chr10 |  | * |  |  |
| CBS408 | chr11 |  | * |  |  |
| P11S130 | chr11 |  |  | * |  |
| P11S164 | chr11 | * |  |  | * |

100SW:100-seed weight; SL:seed length; SWI: seed width; SH:seed height.“*”indicated significance association inlevel a=0.05.

**Table S5 Significantly associated markers for seed traits identified with MLM+Q+K model**

| Traits | Marker name | Chromosome | Environment | p_Marker | Rsq_marker |
| --- | --- | --- | --- | --- | --- |
| 100SW | CBS83 | chr3 | 2016HA | 8.19E-05 | 0.0295 |
|  | CBS162 | chr5 | 2015BJ | 7.85E-05 | 0.0167 |
|  | P7S191 | chr7 | 2015HA | 2.85E-04 | 0.0121 |
|  | CBS381 | chr10 | 2015HA | 3.26E-04 | 0.0154 |
| SL | CBS162 | chr5 | 2015HA | 6.00E-05 | 0.0168 |
|  |  |  | 2015BJ | 4.27E-06 | 0.0199 |
|  | CBS170 | chr5 | 2016HA | 2.23E-04 | 0.0065 |
|  | CBS178 | chr5 | 2014HA | 2.10E-07 | 0.0229 |
|  |  |  | 2015BJ | 2.42E-04 | 0.0089 |
|  | CBS179 | chr5 | 2016BJ | 6.26E-05 | 0.0081 |
| SWI | CBS13 | chr1 | 2014BJ | 2.34E-04 | 0.0236 |
|  | CBS28 | chr1 | 2016BJ | 2.48E-04 | 0.0136 |
|  | CBS91 | chr3 | 2016BJ | 9.59E-05 | 0.0222 |
|  | CBS149 | chr4 | 2014HA | 2.78E-04 | 0.0157 |
|  |  |  | 2015BJ | 3.68E-04 | 0.0157 |
|  |  |  | 2016HA | 1.65E-04 | 0.0218 |
|  | CBS190 | chr5 | 2014BJ | 9.21E-05 | 0.0189 |
|  | CBS208 | chr6 | 2015HA | 3.02E-04 | 0.0131 |
|  | P7S11 | chr7 | 2016BJ | 6.63E-05 | 0.013 |
|  | CBS345 | chr9 | 2015BJ | 3.22E-05 | 0.0208 |
|  |  |  | 2016HA | 4.6434E-06 | 0.0251 |
|  |  |  | 2016BJ | 7.61E-05 | 0.0159 |
|  | P9S153 | chr9 | 2016BJ | 2.40E-04 | 0.0164 |
| SH | CBS57 | chr2 | 2014HA | 4.12E-05 | 0.0243 |
|  | CBS149 | chr4 | 2014HA | 1.31E-04 | 0.0232 |
|  | CBS176 | chr5 | 2014HA | 1.84E-05 | 0.0233 |
|  | CBS381 | chr10 | 2015BJ | 4.24E-04 | 0.02 |

**Table S6 Summary of signifcant marker-trait associations for frost tolerance repeatedly detected in different environments with two models.**

| **Traits** | **Markers** | **Chr** | **GLM+Q** | | | **MLM+Q+K** | | |
| --- | --- | --- | --- | --- | --- | --- | --- | --- |
|  |  |  | **Environments** | **P value** | **R^2^** | **Environments** | **P value** | **R^2^** |
| **100SW** | CBS83 | chr3 | 2015_HRB | 2.2796E-05 | 0.0753 |  |  |  |
|  |  |  | 2016_HRB | 3.16E-07 | 0.1038 | 2016_HRB | 8.19E-05 | 0.0295 |
|  | CBS162 | chr5 | 2014_HRB | 1.9871E-18 | 0.1263 |  |  |  |
|  |  |  | 2014_BJ | 8.7455E-15 | 0.0929 |  |  |  |
|  |  |  | 2015_HRB | 5.0529E-25 | 0.1793 |  |  |  |
|  |  |  | 2015_BJ | 2.82E-18 | 0.1042 | 2015_BJ | 7.85E-05 | 0.0167 |
|  |  |  | 2016_HRB | 6.69E-15 | 0.1494 |  |  |  |
|  |  |  | 2016_BJ | 1.93E-15 | 0.1043 |  |  |  |
|  | P7S191 | chr7 | 2014_HRB | 9.8351E-09 | 0.0640 |  |  |  |
|  |  |  | 2015_HRB | 7.3715E-15 | 0.1104 | 2015_HRB | 2.85E-04 | 0.0121 |
|  |  |  | 2015_BJ | 2.26E-09 | 0.0565 |  |  |  |
|  |  |  | 2016_HRB | 3.52E-13 | 0.1191 |  |  |  |
|  |  |  | 2016_BJ | 6.43E-13 | 0.0794 |  |  |  |
|  | CBS381 | chr10 | 2014_HRB | 1.8815E-19 | 0.1305 |  |  |  |
|  |  |  | 2014_BJ | 5.5462E-11 | 0.0748 |  |  |  |
|  |  |  | 2015_HRB | 3.9004E-24 | 0.1739 | 2015_HRB | 3.26E-04 | 0.0154 |
|  |  |  | 2015_BJ | 4.61E-14 | 0.0857 |  |  |  |
|  |  |  | 2016_HRB | 2.59E-12 | 0.1294 |  |  |  |
|  |  |  | 2016_BJ | 1.36E-13 | 0.0925 |  |  |  |
| **SL** | CBS162 | chr5 | 2014_HRB | 6.8293E-28 | 0.2174 |  |  |  |
|  |  |  | 2014_BJ | 1.6832E-29 | 0.2194 |  |  |  |
|  |  |  | 2015_HRB | 9.6436E-40 | 0.2887 | 2015_HRB | 6.00E-05 | 0.0168 |
|  |  |  | 2015_BJ | 3.18E-37 | 0.2553 | 2015_BJ | 4.27E-06 | 0.0199 |
|  |  |  | 2016_HRB | 2.20E-32 | 0.2974 |  |  |  |
|  |  |  | 2016_BJ | 8.01E-34 | 0.2382 |  |  |  |
|  | CBS170 | chr5 | 2014_HRB | 3.7241E-06 | 0.0333 |  |  |  |
|  |  |  | 2014_BJ | 0.00031803 | 0.0198 |  |  |  |
|  |  |  | 2015_HRB | 0.00022113 | 0.0227 |  |  |  |
|  |  |  | 2015_BJ | 3.77E-05 | 0.0258 |  |  |  |
|  |  |  | 2016_HRB | 1.42E-04 | 0.0280 | 2016_HRB | 2.23E-04 | 0.0065 |
|  |  |  | 2016_BJ | 6.12E-05 | 0.0242 |  |  |  |
|  | CBS178 | chr5 | 2014_HRB | 2.2983E-18 | 0.1536 | 2014_HRB | 2.10E-07 | 0.0229 |
|  |  |  | 2014_BJ | 4.5377E-24 | 0.1853 |  |  |  |
|  |  |  | 2015_HRB | 9.4306E-22 | 0.1843 |  |  |  |
|  |  |  | 2015_BJ | 1.83E-22 | 0.1718 |  |  |  |
|  |  |  | 2016_HRB | 5.57E-24 | 0.2264 |  |  |  |
|  |  |  | 2016_BJ | 2.31E-23 | 0.1768 | 2016_BJ | 2.42E-04 | 0.0089 |
|  | CBS179 | chr5 | 2014_HRB | 1.314E-09 | 0.0713 | 2016_BJ | 6.26E-05 | 0.0081 |
|  |  |  | 2014_BJ | 1.2557E-11 | 0.0821 |  |  |  |
|  |  |  | 2015_HRB | 8.0472E-13 | 0.0977 |  |  |  |
|  |  |  | 2015_BJ | 6.09E-13 | 0.0905 |  |  |  |
|  |  |  | 2016_HRB | 4.91E-13 | 0.1156 |  |  |  |
|  |  |  | 2016_BJ | 1.66E-14 | 0.0994 | 2016_BJ | 6.26E-05 | 0.0081 |
| **SWI** | CBS13 | chr1 | 2014_HRB | 2.9487E-05 | 0.0574 |  |  |  |
|  |  |  | 2014_BJ | 3.8754E-08 | 0.0801 | 2014_BJ | 2.34E-04 | 0.0236 |
|  |  |  | 2015_HRB | 2.5697E-05 | 0.0610 |  |  |  |
|  |  |  | 2016_BJ | 4.21E-05 | 0.0547 |  |  |  |
|  | CBS28 | chr1 | 2014_HRB | 3.6734E-12 | 0.1057 |  |  |  |
|  |  |  | 2014_BJ | 0.00015527 | 0.0467 |  |  |  |
|  |  |  | 2015_HRB | 1.8586E-11 | 0.1060 |  |  |  |
|  |  |  | 2015_BJ | 2.98E-11 | 0.0860 |  |  |  |
|  |  |  | 2016_HRB | 4.14E-16 | 0.1671 |  |  |  |
|  |  |  | 2016_BJ | 3.29E-16 | 0.1275 | 2016_BJ | 2.48E-04 | 0.0136 |
|  | CBS91 | chr3 | 2014_HRB | 2.0631E-15 | 0.1609 |  |  |  |
|  |  |  | 2014_BJ | 9.0588E-07 | 0.0910 |  |  |  |
|  |  |  | 2015_HRB | 9.8567E-14 | 0.1565 |  |  |  |
|  |  |  | 2015_BJ | 3.52E-15 | 0.1399 |  |  |  |
|  |  |  | 2016_HRB | 2.15E-13 | 0.1812 |  |  |  |
|  |  |  | 2016_BJ | 1.43E-21 | 0.1920 | 2016_BJ | 9.59E-05 | 0.0222 |
|  | CBS149 | chr4 | 2014_HRB | 1.1882E-20 | 0.1694 | 2014_HRB | 2.78E-04 | 0.0157 |
|  |  |  | 2014_BJ | 2.9568E-08 | 0.0776 |  |  |  |
|  |  |  | 2015_HRB | 1.52E-17 | 0.1545 |  |  |  |
|  |  |  | 2015_BJ | 6.17E-19 | 0.1386 | 2015_BJ | 3.68E-04 | 0.0157 |
|  |  |  | 2016_HRB | 1.01E-21 | 0.2157 | 2016_HRB | 1.65E-04 | 0.0218 |
|  |  |  | 2016_BJ | 1.91E-20 | 0.1615 |  |  |  |
|  | CBS190 | chr5 | 2014_HRB | 9.7779E-06 | 0.0467 | 2014_BJ | 9.21E-05 | 0.0189 |
|  | CBS208 | chr6 | 2015_HRB | 4.1182E-05 | 0.0528 | 2015_HRB | 3.02E-04 | 0.0131 |
|  | P7S11 | chr7 | 2015_BJ | 1.22E-05 | 0.0394 | 2015_BJ | 6.63E-05 | 0.0130 |
|  | *Phs* | chr7 | 2015_BJ | 4.86E-05 | 0.0538 | 2015_BJ | 1.39E-05 | 0.0203 |
|  |  |  | 2016_BJ | 4.43E-06 | 0.0676 |  |  |  |
|  | CBS345 | chr9 | 2014_HRB | 2.1899E-15 | 0.1314 |  |  |  |
|  |  |  | 2014_BJ | 1.3676E-06 | 0.0654 |  |  |  |
|  |  |  | 2015_HRB | 6.8517E-15 | 0.1348 |  |  |  |
|  |  |  | 2015_BJ | 4.48E-18 | 0.1330 | 2015_BJ | 3.22E-05 | 0.0208 |
|  |  |  | 2016_HRB | 1.01E-19 | 0.1965 | 2016_HRB | 4.64E-06 | 0.0251 |
|  |  |  | 2016_BJ | 3.96E-22 | 0.1709 | 2016_BJ | 7.61E-05 | 0.0159 |
|  | P9S153 | chr9 | 2014_HRB | 1.09E-17 | 0.1569 |  |  |  |
|  |  |  | 2014_BJ | 7.5673E-08 | 0.0865 |  |  |  |
|  |  |  | 2015_HRB | 1.9184E-12 | 0.1284 |  |  |  |
|  |  |  | 2015_BJ | 1.93E-17 | 0.1405 |  |  |  |
|  |  |  | 2016_HRB | 2.31E-17 | 0.1943 |  |  |  |
|  |  |  | 2016_BJ | 7.82E-23 | 0.1840 | 2016_BJ | 2.40E-04 | 0.0164 |
| **SH** | CBS57 | chr2 | 2014_HRB | 2.65E-14 | 0.1282 | 2014_HRB | 4.12E-05 | 0.0243 |
|  |  |  | 2014_BJ | 5.03E-09 | 0.0822 |  |  |  |
|  |  |  | 2015_HRB | 5.9652E-12 | 0.1106 |  |  |  |
|  |  |  | 2015_BJ | 5.65E-13 | 0.1151 |  |  |  |
|  |  |  | 2016_HRB | 3.51E-12 | 0.1391 |  |  |  |
|  |  |  | 2016_BJ | 1.48E-12 | 0.1085 |  |  |  |
|  | CBS149 | chr4 | 2014_HRB | 1.2174E-10 | 0.1103 | 2014_HRB | 1.31E-04 | 0.0232 |
|  |  |  | 2014_BJ | 2.2946E-07 | 0.0756 |  |  |  |
|  |  |  | 2015_HRB | 5.9652E-12 | 0.1106 |  |  |  |
|  |  |  | 2015_BJ | 1.23E-09 | 0.0978 |  |  |  |
|  |  |  | 2016_HRB | 1.02E-11 | 0.1459 |  |  |  |
|  |  |  | 2016_BJ | 4.65E-10 | 0.0997 |  |  |  |
|  | CBS381 | chr10 | 2014_HRB | 8.4667E-14 | 0.1611 |  |  |  |
|  |  |  | 2014_BJ | 1.7378E-12 | 0.1426 |  |  |  |
|  |  |  | 2015_HRB | 5.3194E-21 | 0.2151 |  |  |  |
|  |  |  | 2015_BJ | 1.42E-17 | 0.1849 | 2015_BJ | 4.24E-04 | 0.0200 |
|  |  |  | 2016_HRB | 1.39E-18 | 0.2449 |  |  |  |
|  |  |  | 2016_BJ | 1.77E-13 | 0.1503 |  |  |  |

All the results with a strict threshold of 4.3×10-4 at a significance level of 0.05 after Bonferroni correction in both models.

**Supplementary Figure 1.** The variation of phaseolin in 395 accessions. Eleven phaseolin patterns were identified among these accessions: S, Sb, Sd, B, C, CA,CH, H, PA, T, To. Among these, S, Sb, Sd, B belong to Mesoamerican types while C, CA,CH, H, PA, T, To were belonged to Andean types. Different color indicated different type of phaseolin and the figure means percentage of each phaseoiln type. Sb and T was the predominance types of Mesoamerican and Andean genepool, respectively.

**Supplementary Figure 2.** Distribution of different germplasm categories in two major groups. Landrace-China and Modern Cultivars-China indicated landrace of common bean in China and modern breeding cultivars in China, respectively.Non-China mean the accessions introduced from from abroad.
